# CDC48A, an interactor of WOX2, is required for embryonic patterning in *Arabidopsis thaliana*

**DOI:** 10.1007/s00299-024-03158-2

**Published:** 2024-06-15

**Authors:** Wen Gong, Deniz Tiambeng Bak, Jos R. Wendrich, Dolf Weijers, Thomas Laux

**Affiliations:** 1 Present Address: Institute of Plant Sciences, University of Regensburg, Universitätsstraße 31, 93053 Regensburg, Germany; 2 Wageningen University, 6703 Wageningen, The Netherlands; 3 Signalling Research Centres BIOSS and CIBSS, Faculty of Biology, University of Freiburg, Schänzlestrasse 1, 79104 Freiburg, Germany

**Keywords:** Embryogenesis, Shoot meristem, *Arabidopsis thaliana*, WOX2, CDC48A

## Abstract

**Key message:**

Interactor of WOX2, CDC48A, is crucial for early embryo patterning and shoot meristem stem cell initiation, but is not required for WOX2 protein turnover or subcellular localization.

**Abstract:**

During *Arabidopsis* embryo patterning, the WUSCHEL HOMEOBOX 2 (WOX2) transcription factor is a major regulator of protoderm and shoot stem cell initiation. Loss of WOX2 function results in aberrant protodermal cell divisions and, redundantly with its paralogs WOX1, WOX3, and WOX5, compromised shoot meristem formation. To elucidate the molecular basis for WOX2 function, we searched for protein interactors by IP–MS/MS from WOX2-overexpression roots displaying reprogramming toward shoot-like cell fates. Here, we report that WOX2 directly interacts with the type II AAA ATPase molecular chaperone CELL DIVISION CYCLE 48A (CDC48A). We confirmed this interaction with bimolecular fluorescence complementation and co-immunoprecipitation and found that both proteins co-localize in the nucleus. We show that *CDC48A* loss of function results in protoderm and shoot meristem stem cell initiation defects similar to *WOX2* loss of function. We also provide evidence that CDC48A promotes WOX2 activity independently of proteolysis or the regulation of nuclear localization, common mechanisms of CDC48A function in other processes. Our results point to a new role of CDC48A in potentiating WOX2 function during early embryo patterning.

**Supplementary Information:**

The online version contains supplementary material available at 10.1007/s00299-024-03158-2.

## Introduction

The basic body plan of the embryo in *Arabidopsis thaliana* (hereafter *Arabidopsis*) is established during early embryogenesis. At the eight-cell stage, four groups of cells have formed with different developmental fates along the apical–basal embryo axis: the apical domain gives rise to the shoot meristem and cotyledons, the central domain to the hypocotyl and root, the hypophyseal cell to the distal root meristem, and the suspensor connects the embryo to the maternal tissue. At the 16-cell stage, the protoderm is separated from the inner cells by a round of periclinal cell divisions, setting up the radial (inner–outer) embryo axis. During subsequent developmental stages, cells acquire their developmental fate based on their spatial position. At the shoot and root poles, the primordia of the apical meristems are formed, which will give rise to the cells required for continuous plant growth after germination (Bäurle and Laux 2003; Capron et al. 2009; Laux et al. 2004; Yoshida et al. 2014).

WOX2 belongs to the youngest evolutionary clade of the WUSCHEL RELATED HOMEOBOX (WOX) family of transcription factors, many of which regulate stem cell and embryo development in angiosperms (Haecker et al. 2004; van der Graaff et al. 2009). Expression of the *WOX2* gene commences in the zygote, is successively confined to the apical protoderm of the embryo, and terminates just before the shoot meristem becomes apparent (Haecker et al. 2004). *wox2* loss-of-function embryos display patterning defects at different stages. Among these defects are abnormal anticlinal cell divisions at the eight-cell stage, resulting in incomplete protoderm formation. From the globular stage onwards, *WOX2* promotes the formation of a cytokinin domain in the center of the apical embryo and is required for the timely initiation of the shoot meristem and expression of the stem cell marker *CLAVATA3* (*CLV3)* (Zhang et al. 2017). These defects are enhanced in the *wox1-2 wox2-1 wox3-2 wox5-1* (*wox1235)* quadruple mutant (Breuninger et al. 2008). However, genetic analyses indicate that *WOX2* is a major regulator in embryo patterning, whereas *WOX1*, *WOX3*, and *WOX5* seem to take over redundant functions only if *WOX2* is non-functional.

Once initiated, the maintenance of the shoot meristem is governed by the WOX2 paralog and founding member of the WOX family, the *WUSCHEL* (*WUS*) gene. *WUS* is expressed in the organizing center (OC) of the shoot meristem from where it maintains the overlying stem cell pool and directly promotes the expression of the negative feedback signal CLV3 (Lenhard and Laux 2003; Schoof et al. 2000; Yadav et al. 2011). In the embryo, *WUS* expression commences in the precursor cells of the OC at the 16-cell stage. Still, it is neither sufficient nor required for stem cell initiation, as indicated by the timely onset of *CLV3* expression at the heart stage of embryogenesis (Mayer et al. 1998; Tucker et al. 2008; Zhang et al. 2017).

It is unclear why WOX2 can initiate shoot meristem formation but not WUS, although WUS is already expressed in the precursor cells of the OC, and WOX2 can partially substitute for WUS in postembryonic stem cell maintenance (Dolzblasz et al. 2016). Both proteins contain a conserved homeodomain and a WUS-box, but WUS also contains acidic and EAR domains not found in WOX2 (van der Graaff et al. 2009), suggesting, at least in part, different molecular workings of both proteins.

CELL DIVISION CYCLE 48 (CDC48) (with homologs CDC48p in yeast and p97/vasolin-containing protein in mammals) has been found in many organisms to be involved in various cellular processes, including cellular signaling, protein degradation, vesicular trafficking, and autophagy (Baek et al. 2013; Begue et al. 2017). The active form of CDC48 is a barrel-shaped homohexameric complex using energy from ATP hydrolysis to modify proteins. CDC48 can function as a ubiquitin-dependent unfoldase/segregase and requires interaction with diverse cofactors for substrate selection (Bodnar and Rapoport 2017; Meyer et al. 2012; Ramadan et al. 2017). Although homologs of *CDC48* have been identified in a few plant phylogenetic clades (Begue et al. 2017), functional studies of CDC48 have been majorly conducted in *Arabidopsis,* where five CDC48 paralogs exist: A (AT3G09840), B (AT3G53230), C (AT5G03340), D (AT2G03670), and E (AT3G01610) (Copeland et al. 2016), all of which are expressed in the embryo (Hofmann et al. 2019). While most studies have been carried out on CDC48A, very few have identified the functions of the other paralogs in *Arabidopsis*, albeit in other contexts (Niehl et al. 2012; Yang et al. 2022).

We searched for interacting proteins to gain insight into the molecular mechanism by which WOX2 functions in early patterning. Here, we report that CDC48A is a direct interactor of WOX2 and is required for protoderm formation and shoot meristem development. Our results further suggest that CDC48A potentiates WOX2 activity during embryo patterning, independently of its canonical functions in protein degradation or nuclear localization of client proteins, suggesting a previously unreported function of CDC48A.

## Results

### WOX2 interacts with the AAA^+^ ATPase CDC48A ***in planta***

**Fig. 1:**
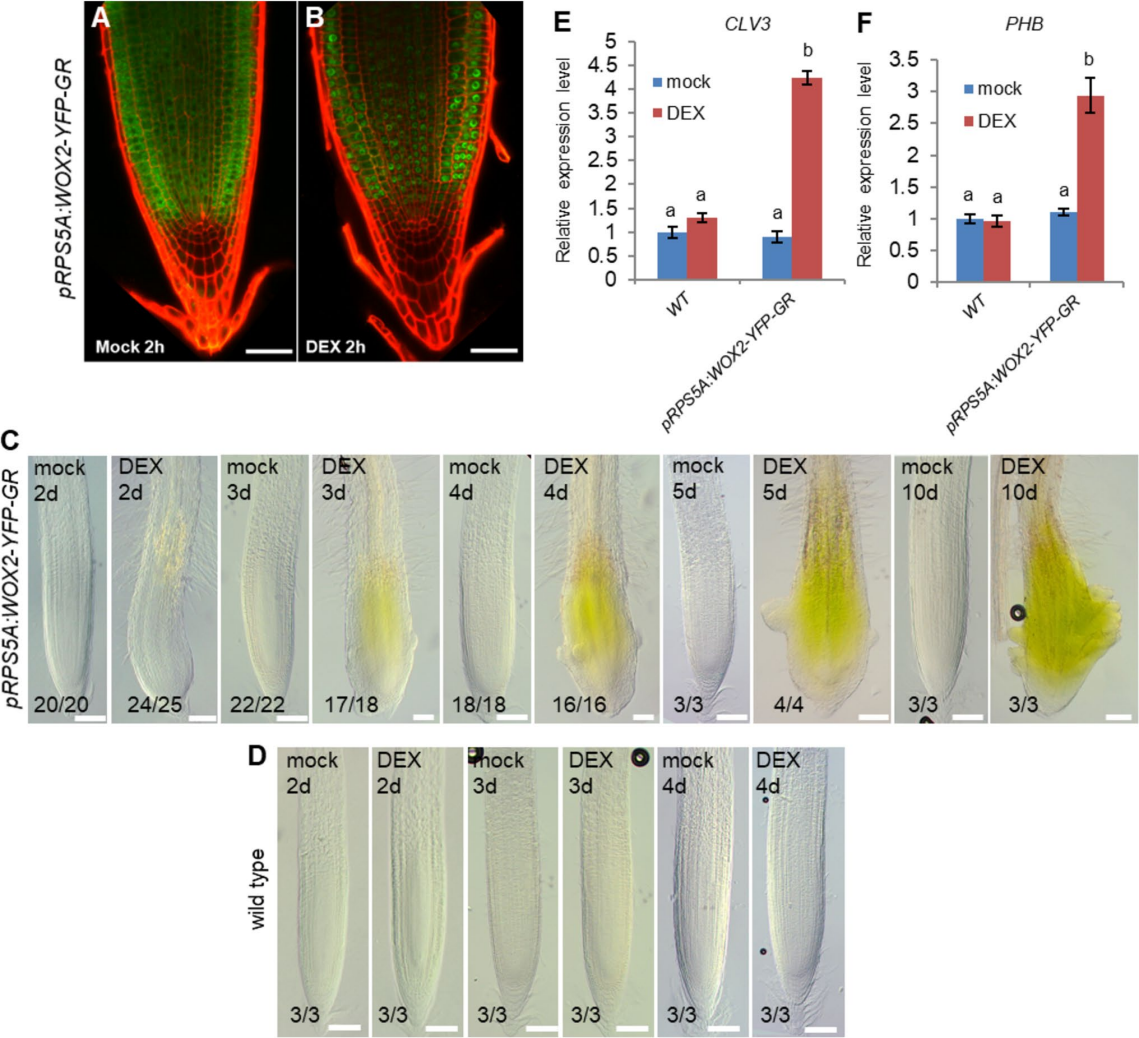
Ectopic WOX2 expression in the root leads to over-proliferation and expression of shoot-like traits. a and **b** Expression of *pRPS5A:WOX2-YFP-GR* (green) in the wild-type background of roots grown for 5 days on ½MS with 2 h mock treatment (**a**) or ½MS with 2 h 10 μM DEX treatment (**b**). Cell walls were stained by propidium iodide (red). *n* > 15 for each treatment. Scale bars: 50 μm. **c** and **d** Root phenotypes of *pRPS5A:WOX2-YFP-GR* (**c**) and wild-type (**d**) seedlings treated with either mock or 10 μM DEX from 2 to 10 days as indicated. Scale bars: 100 μm. **e** and **f** Relative expression levels of *CLV3* (**e**) and *PHB* (**f**) from the roots of 6-day-old (4 days on MS with 2 days on 10 μM DEX or mock treatment) wild type (WT) and *pRPS5A:WOX2-YFP-GR*. Data represents mean values ± SD of three biological replicates by quantitative RT-PCR normalized to the internal control (*TIP41*). **a** and **b** indicate significance categories (*p* < 0.001) by one-way ANOVA with a post hoc Tukey–Kramer test

**Fig. 2:**
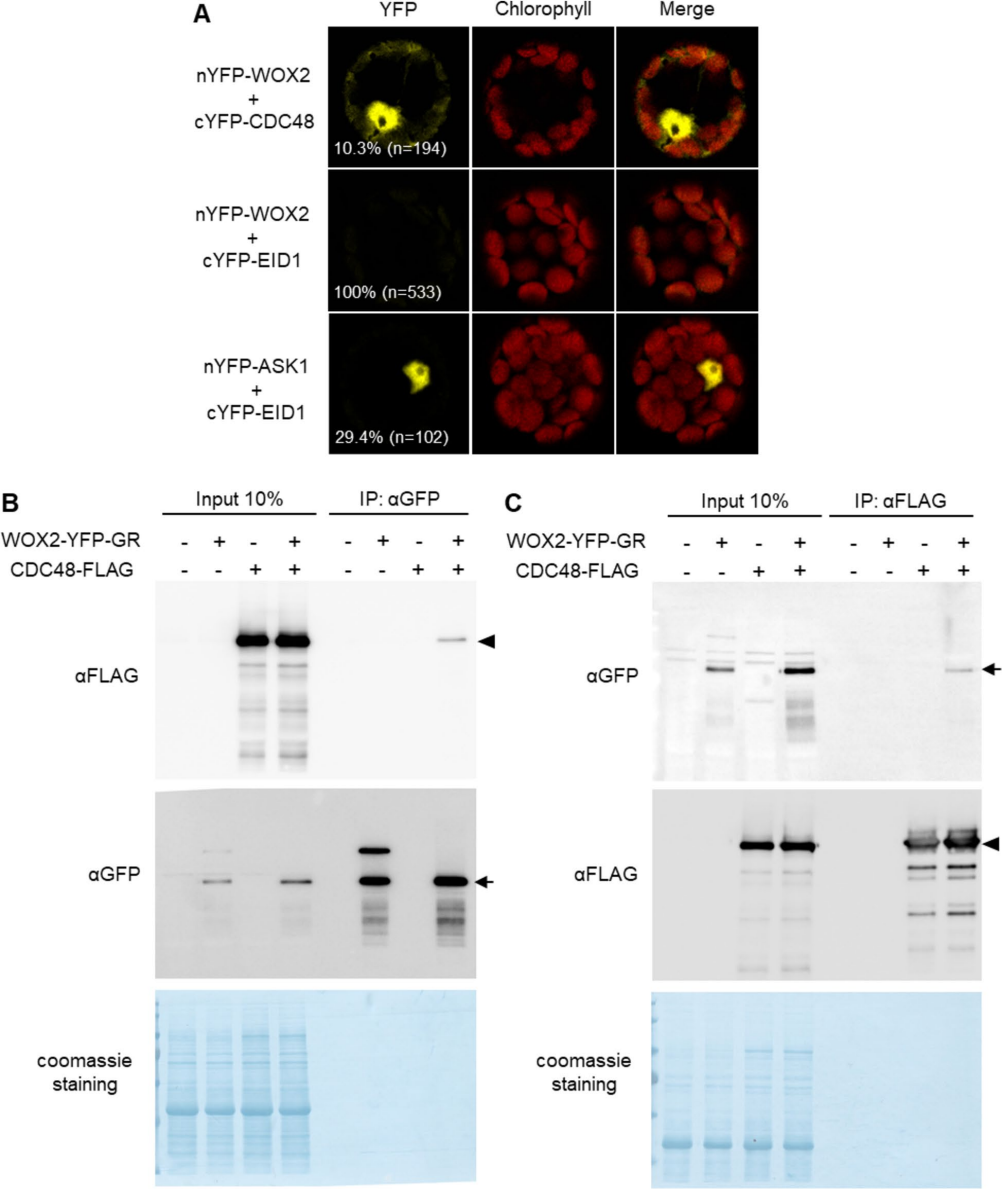
CDC48A interacts with WOX2 in plantabb. a Confocal microscopy images of a BiFC assay showing interaction between WOX2 and CDC48A proteins in *Arabidopsis thaliana* leaf mesophyll protoplasts. The negative control shows no interaction between WOX2 and EID1. As a positive control, EID1 and ASK1 showed interaction with each other. The percentages of protoplasts with YFP signal and the numbers of total protoplasts observed are indicated. Scale bars: 20 μm. **b** and **c** Co-IP results from wild type, *pRPS5a:WOX2-YFP-GR*, *p35S:CDC48A-FLAG* and *p35S:CDC48A-FLAG/ pRPS5a:WOX2-YFP-GR*. Proteins extracted from 5-day-old seedlings after a 4-h 10 μM DEX induction were immunoprecipitated by anti-GFP beads (**b**) or anti-FLAG beads (**c**). Western blots using anti-GFP and anti-FLAG to detect WOX2 and CDC48A, respectively, are shown. Arrowheads indicate CDC48A-FLAG, and arrows indicate WOX2-YFP-GR. Coomassie blue staining as loading controls are shown

To investigate the molecular mechanism of WOX2 function in early embryo patterning and stem cell initiation (Zhang et al. 2017), we searched for proteins interacting with WOX2 by IP–MS/MS. Because obtaining sufficient protein from early-stage embryos is difficult, we asked whether ectopically expressing *WOX2* in root cells would induce shoot meristem stem cell identity, as previously reported for its close paralog *WUS* (Gallois et al. 2004) and thus enable us to extract WOX2 protein interactors from reprogrammed root cells as a proxy. To circumvent potential detrimental effects by constitutively overexpressing WOX2, we generated plants with WOX2-YFP fused to a glucocorticoid receptor (GR) (Aoyama and Chua 1997) driven by the ubiquitous *RIBOSOMAL PROTEIN SUBUNIT 5A* (*RPS5A)* promoter (Weijers et al. 2001). Upon treating *pRPS5A*:*WOX2-YFP-GR* roots with 10-μM DEX for 2 h, we found that WOX2-YFP-GR completely shifts from the cytoplasm into nuclei of the root cells (Fig. 1a, b). 2 days after DEX induction, the roots of 6-day-old seedlings expanded in width and generated more root hairs. 3–4 days of DEX treatment resulted in the roots greening at the meristematic region and bulges protruding from the root surface. After 5–10 days of DEX treatment, the bulges were also present in the transition and differentiation zones. The bulges did not develop into lateral roots but into a central dome surrounded by several primordia-like protrusions reminiscent of a shoot meristem surrounded by leaf primordia (Fig. 1c). Furthermore, a qRT-PCR showed that a 2-day incubation with DEX caused upregulated expression of the shoot stem cell marker *CLV3* (Fig. 1e, Table S1) and the *WOX2* effector gene in shoot meristem initiation, *PHABULOSA (PHB)* (Fig. 1f, Table S1). In contrast, wild-type roots showed no phenotypic abnormalities or altered relative expression levels of *CLV3* and *PHB* after treatment with DEX (Fig. 1d–f, Table S1). These observations suggest that ectopic WOX2 activity reprograms root cells toward at least some aspects of shoot meristem formation. Therefore, we used the roots of DEX-induced *pRPS5A:WOX2-YFP-GR* as a proxy for *WOX2* transcriptional activity in shoot meristem initiation and searched for interactors. Since, at this time, *WOX2-YFP-GR* has moved into the nucleus, we harvested roots after a 4-h DEX induction, followed by an IP–MS/MS using an anti-GFP antibody. We hypothesized that this strategy would preferentially identify proteins interacting with WOX2 in the nucleus, where it plausibly executes its regulatory function. From several enriched proteins (Table S2), we focused on CDC48A for further analysis because *cdc48a* T-DNA insertion mutants have been reported to display embryo development defects (Park et al. 2008). We confirmed the interaction between CDC48A and WOX2 by bimolecular fluorescence complementation (BiFC) in *Arabidopsis* mesophyll protoplasts (Fig. 2a) and by co-immunoprecipitation from *Arabidopsis* plants stably expressing WOX2-YFP-GR and CDC48-FLAG proteins (Fig. 2b, c). Furthermore, by analyzing different truncated variants, we showed that the N-terminal domain of CDC48A is required for interaction with WOX2 (Fig. S1a, b), consistent with previous reports showing that the N-terminal domain of CDC48 is responsible for binding to cofactors and substrates (Park et al. 2007).

### CDC48A knockout mutants are defective in embryonic protoderm and shoot meristem formation, similar to the *WOX2* loss-of-function mutants

Previous reports have shown that *wox2* mutant embryos fail to establish a contiguous protoderm at the 16-cell stage due to abnormal cell divisions (Breuninger et al. 2008) and to initiate shoot meristem stem cells at the early heart stage, and these defects are strongly enhanced in the *wox1-2 wox2-4 wox3-2 wox5-1* (*wox1235*) quadruple mutant (Zhang et al. 2017). To address whether the loss of CDC48A function causes similar defects, we studied three T-DNA insertion mutant lines, *cdc48a-1, cdc48a-2,* and *cdc48a-3*, where the T-DNA is inserted in the first intron, third exon, and third intron of *CDC48A*, respectively (Park et al. 2008). All three T-DNA insertion sites are upstream of the sequence encoding the two ATPase domains, and all three segregating T-DNA insertion mutants exhibited the same frequency of embryos with protoderm defects at the 16-cell stage (Fig. S2a–d). This strongly suggests that the observed defects were due to a lack of CDC48A function. We focused on *cdc48a-1* for a detailed phenotypic analysis.

**Fig. 3:**
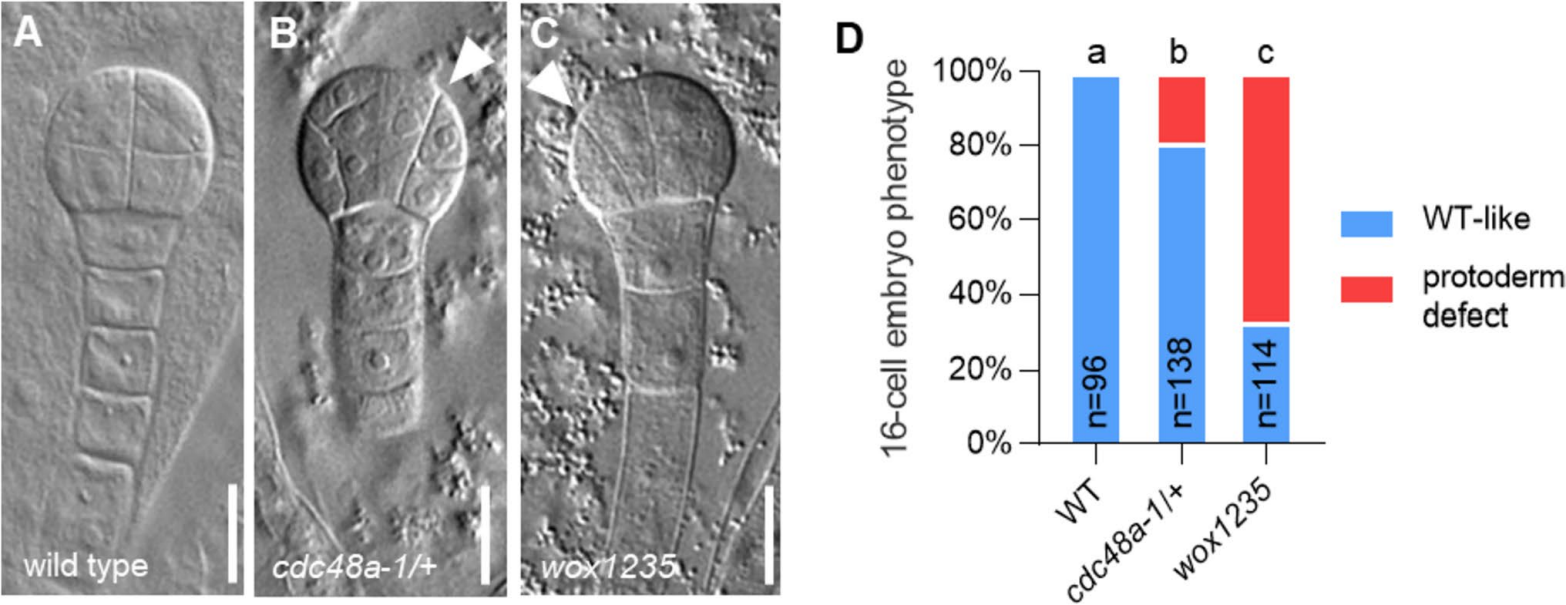
CDC48A loss of function shows defects in protoderm formation. a–**c** Phenotypes of 16-cell stage embryos from wild-type (**a**), *wox1235* (**b**), and *cdc48a-1/*+ (**c**) mother plants. White arrowheads point to the abnormal cell division plane. Scale bar: 10 μm. **d** Frequencies of cell division patterning phenotypes from 16-cell stage embryos from wild-type (WT), *cdc48a-1/*+, and *wox1235* mother plants. **a**, **b**, and **c** indicate significance categories (*p* < 0.0001) by Fisher’s exact test with Bonferroni correction

Unlike the wild-type embryos that undergo a round of periclinal cell divisions at the eight-cell stage that separates the outer protodermal layer from the inner cells (Fig. 3a, d), 19% (*n* = 138) of the segregating embryos from a *cdc48a-1/*+ mother do not display anticlinal divisions and, consequently, display a disrupted apical protodermal layer (Fig. 3b, d). Notably, these defects are very similar to those in the *wox1235* mutant (Fig. 3c, d). Thus, *CDC48A* and *WOX2* appear to play similar roles in embryonic protoderm formation.

**Fig. 4:**
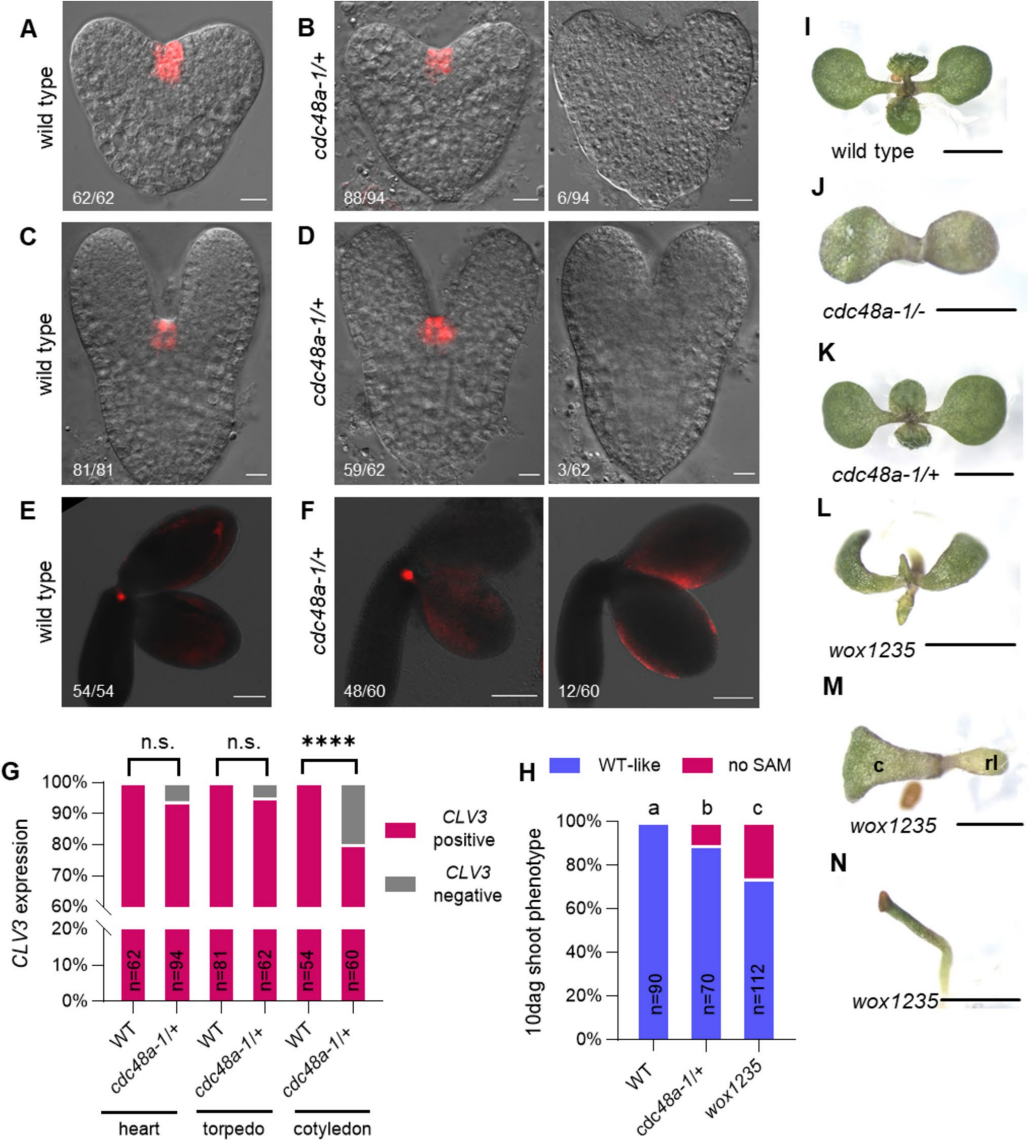
CDC48A is necessary for shoot meristem development. a–f *pCLV3:er-tdTomato* expression in heart (**a,** **b**), torpedo (**c** and **d**), and bent-cotyledon (**e,** **f**) stage embryos from mother plants of the indicated genotypes. Scale bars: 20 μm. **g** Frequencies of embryos with and without *pCLV3:er-tdTomato* expression. WT, wild type; n.s. not significant, ****, *p* < 0.0001 (Fisher’s exact test). **h** Frequencies of 10-day-old seedlings with or without a SAM. WT, wild type. **a**, **b**, and **c** indicate significance categories (*p* < 0.01) by Fisher’s exact test with Bonferroni correction. **i–n** Representative images of 10-day-old seedling phenotypes of the indicated genotypes. The functional shoot apical meristem (SAM) is present in the wild type (**i**) and *cdc48a-1/*+ (**k**). Terminated SAM in *cdc48a-1*/- (**j**). *wox1235* shows wild type-like shoot with a functional SAM (**l**), or a single cotyledon (c) with a SAM and one rosette leaf (rl) (**m**), or a pin-like shoot structure (**n**). Scale bars: 2 mm

We next investigated shoot meristem initiation, monitoring the expression of the stem cell reporter gene *pCLV3:er-tdTomato* (Zhang et al. 2017). We detected *pCLV3:er-tdTomato* in 100% wild-type embryos at the heart stage (Fig. 4a, g), the late heart-to-torpedo stages (Fig. 4c, g), and the bent-cotyledon stage (Fig. 4e, g). By contrast, an increasing fraction of the segregating *cdc48a-1/*+ embryos lacked *CLV3* reporter expression during progressive development: 6.4% at the heart stage (Fig. 4b, g), 4.8% at late heart-torpedo stages (Fig. 4d, g), and 20% at the bent-cotyledon stage (Fig. 4f, g). Furthermore, *pCLV3:er-tdTomato* expression levels were weaker than in the wild type in the remaining embryos (Fig. S3). These observations suggest that CDC48A is required for the timely initiation and normal levels of embryonic *CLV3* expression.

We then examined the phenotypes of 10-day-old seedlings from a *cdc48a-1/*+ mother plant and PCR-genotyped 10.3% of the seedlings (*n* = 78) as homozygous *cdc48a-1*, 50% as *cdc48a-1/*+*,* and 32.0% as wild type, with the remaining 7.7% of the seeds not germinating (Table S3). The homozygous *cdc48a-1* seedlings displayed a terminated shoot apical meristem without producing any leaves (Fig. 4h, j), a short root, and failed to grow further after (data not shown). The heterozygous *cdc48a-1/*+ seedlings showed normal root and vegetative shoot growth (Fig. 4h, k). In comparison, around 26% (*n* = 112) of 10-day-old *wox1235* seedlings exhibited no visible shoot meristem (Fig. 4h, m, and n), consistent with previous findings (Zhang et al. 2017).

**Fig. 5:**
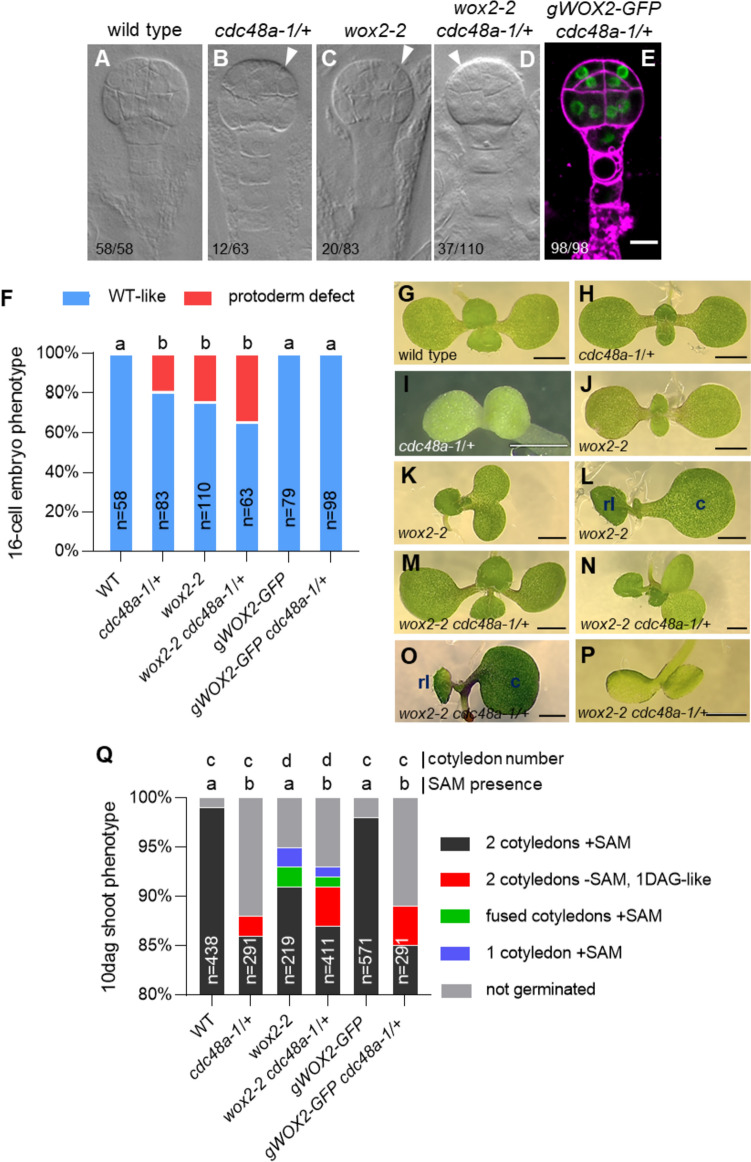
CDC48A and WOX2 function in the same process. a–e 16-cell stage embryos from mother plants of the indicated genotypes. White arrowheads point to abnormal cell division planes. FM4-64 counterstaining of the plasma membranes is shown (magenta). Scale bar: 10 μm. **f** Frequencies of embryo phenotypes at the 16-cell embryo stage of the indicated genotypes. WT, wild type. a and b indicate significance categories (*p* < 0.001) by Fisher’s exact test with Bonferroni correction. **g–p** Representative images of 10-day-old shoot phenotypes of the wild type (G), *cdc48a-1*/+ (**h**–**i**), *wox2-2* (**j**–**l**), *wox2-2 cdc48a-1*/+ (**m**–**p**). Shoot apical meristem (SAM) absence is observed in *cdc48a-1*/+ (**i**) and *wox2-2 cdc48a-1*/+ (**p**). Fused cotyledons are observed in *wox2-2* (**k**) and *wox2-2 cdc48a-1*/+ (**n**). Single cotyledon (c) with one rosette leaf (rl) is seen in *wox2-2* (**l**) and *wox2-2 cdc48a-1*/+ (**o**). Scale bars: 1 mm. **q** Frequencies of 10-day-old seedling shoot phenotypes in the indicated genotypes. WT, wild type. **a** and **b** indicate significance categories concerning SAM presence (*p* < 0.0001) by Fisher’s exact test with Bonferroni correction. **c** and **d** indicate significance categories concerning cotyledon outgrowth (*p* < 0.01) by Fisher’s exact test with Bonferroni correction. *SAM* shoot apical meristem, *DAG* days after germination

Because the *cdc48a-1*/+ protodermal defects, the delay or absence of *CLV3* expression, and the absence of a functional shoot meristem are very similar to the defects exhibited by *wox2-1* (Breuninger et al. 2008) and *wox1235* (Zhang et al. 2017)*,* we addressed the genetic relationship of *CDC48A* and *WOX2*. Since a *wox1235 cdc48a-1/*+ plant could not be constructed due to close genetic linkage between the *WOX5* and *CDC48A* loci (Table S4), we analyzed the *wox2-2 cdc48a-1*/+ double mutant. We found that the frequency of aberrant protoderm formation at the 16-cell embryo stage was not significantly enhanced compared to *wox2-2* or *cdc48a-1*/+ embryos (Figs. 5a–d and f). Likewise, we did not find any enhanced shoot apical meristem defects in segregating *wox2-2 cdc48a-1*/+ seedlings compared to *cdc48a-1*/+ or *wox2-2* (Table S5, Fig. 5g–q). These findings support the hypothesis that CDC48A and WOX2 genetically act in one pathway in protoderm and shoot meristem formation.

We then asked whether increased WOX2 activity could compensate for the loss of CDC48A by introducing a second copy of WOX2. Indeed, expressing *pWOX2(6.5 kb):GFP-WOX2* (hereafter *gWOX2-GFP*) fully rescued the protoderm defect in *cdc48a-1*/+ 16-cell stage embryos (Fig. 5e, f) and globular stage embryos (Fig. S4d, e). This suggests that CDC48A potentiates the function of physiological levels of WOX2 in protoderm formation. By contrast, *gWOX2-GFP* did not rescue the shoot meristem defects in segregating 10-day-old *cdc48a-1*/+ seedlings (Table S5, Fig. 5q).

**Fig. 6:**
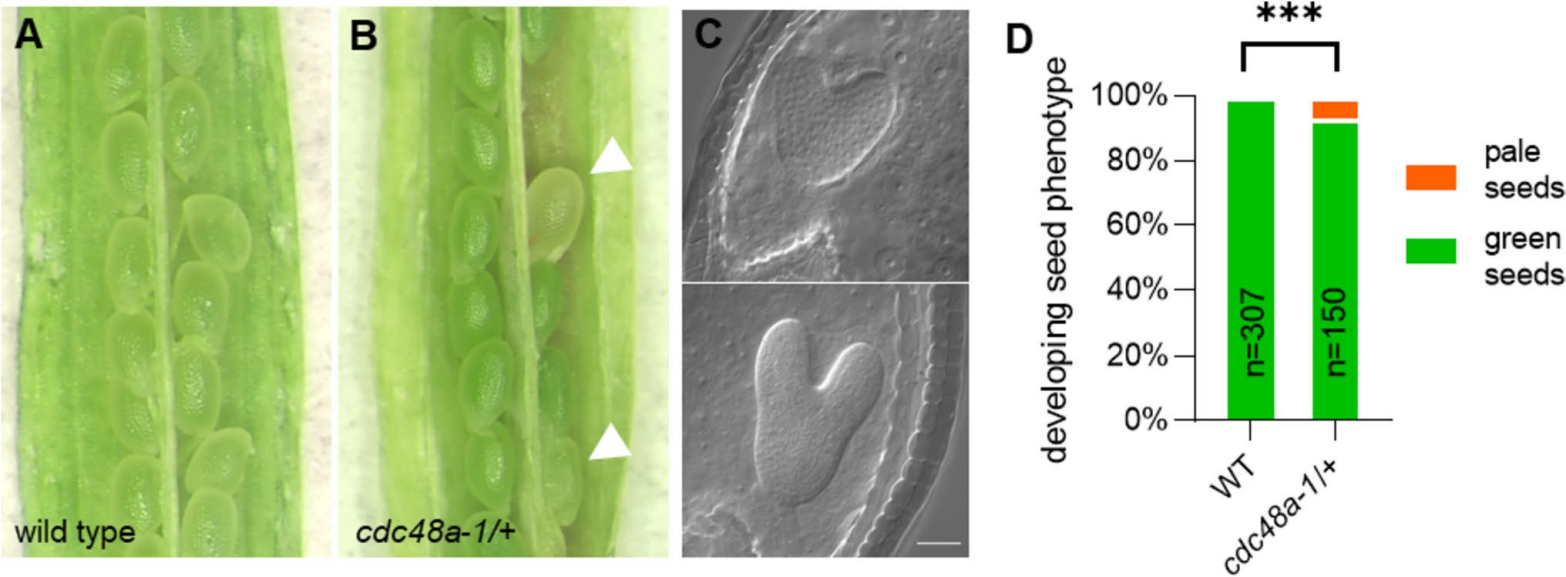
CDC48A loss of function shows defects in timely embryo development. a, **b** Developing seeds of wild type (**a**) and *cdc48a-1/*+ (**b**) in the 15th silique. Pale seeds are indicated by white arrowheads. **c** Pale seeds from the 15th silique contained heart-to-torpedo stage embryos. Scale bar: 20 μm. **d** Frequencies of developing seed phenotypes from wild-type (WT) and *cdc48a-1/*+ 15th siliques. ***, *p* < 0.001 (Fisher’s exact test)

In addition to their shared phenotypes, we found several aberrations in the *cdc48a-1* mutant not observed in *wox2 or wox1235*. First, around 15% of the embryos in a segregating *cdc48a-1* population that initiated *pCLV3:er-tdTomato* expression failed to maintain it (Fig. 4g). This suggests that CDC48A not only plays a role in shoot stem cell initiation, but also in shoot stem cell maintenance. Second, we found a delay in embryo development in a fraction of the segregating *cdc48a-1*/+ (Table S6). In the sixth silique, the majority (43.5%, *n* = 147) of the embryos are at the torpedo stage in a wild-type plant. By contrast, only 0.5% (*n* = 184) of the embryos from a *cdc48a-1/*+ mother plant are at the torpedo stage, whereas the remaining embryos are at the globular to heart stages (Table S6). In the 15th silique, all embryos of a wild-type mother were fully green at the bent-cotyledon stage (Fig. 6a, d, Table S6). By contrast, only a smaller fraction (89.3%, *n* = 150) of embryos from the 15th silique of a *cdc48a-1*/+ mother was at the bent-cotyledon stage, and remaining ovules were pale with embryos at the heart-to-torpedo stages (Fig. 6b–d, Table S6). Third, although the frequencies of *cdc48a-1* seedlings without an apparent shoot meristem varied (Tables S3 and S5), we never observed 25% as expected for segregating homozygotes. Furthermore, we did not observe a significant number of lethal embryos in *cdc48a-1/*+ siliques (Table S3). Therefore, we performed reciprocal crosses and found a reduced paternal transmission of the mutant allele (Table S7). This can explain the underrepresentation of segregating *cdc48a-1* homozygotes, consistent with previous observations (Park et al. 2008).

Thus, in addition to its common functions with *WOX2*, *CDC48A* plays a role in other processes.

### CDC48A co-localizes with WOX2 in embryo nuclei

**Fig. 7:**
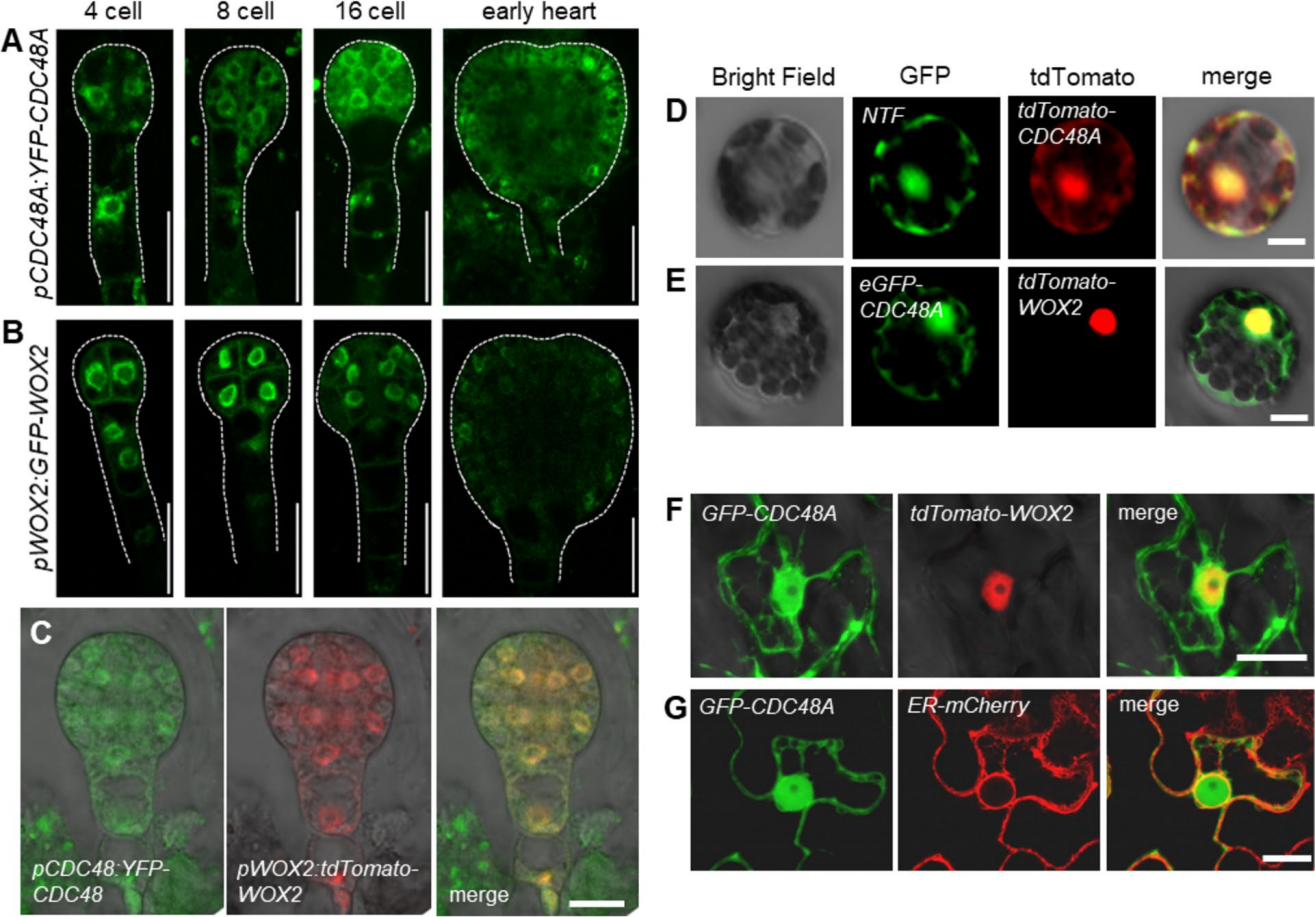
CDC48A co-localizes with WOX2 in the nucleus. a–**c** Representative confocal microscopy images of *pCDC48A:YFP-CDC48A* expression (**a**) and *pWOX2(6.5 kb):GFP-WOX2* expression (**b**) at the indicated embryo stages. *n* > 30 per developmental stage. Scale bars: 20 μm. **c** Co-expression of *pCDC48A:YFP-CDC48A* and *pWOX2(3.5 kb):tdTomato-WOX2* at the globular embryo stage. *n* > 30. Scale bar: 20 μm. **d** and **e** Confocal microscopy images showing co-localization of *35S:tdTomato-CDC48A* and *35S:NTF* (nuclear targeting GF*P* fusion protein) (**d**), and co-localization of *35S:tdTomato-WOX2* and *35S:GFP-CDC48A* (**e**) in *Arabidopsis* protoplasts. (*n* > 30). Scale bars: 20 μm. **f** and **g** Confocal microscopy images showing co-localization of *35S:GFP-CDC48A* and *35S:tdTomato-WOX2* (**f**), and *35S:GFP-CDC48A* and *35S:ER-mCherry* (**g**) in tobacco leaf cells. (*n* > 10). Scale bars: 20 μm

To examine whether CDC48A and WOX2 expression patterns overlap during embryogenesis, we analyzed transgenic plants expressing a YFP-CDC48A fusion protein from its native promoter comprising 754 bp upstream of its transcription start site (Park et al. 2008). *pCDC48A:YFP-CDC48A* fully complements the protodermal cell division patterning defect of the segregating *cdc48a-1*/+ embryos and the shoot apical meristem defect of homozygous *cdc48a-1* seedlings (Fig. S5a, b), indicating that the transgene is functional in these processes. We detected *pCDC48A:YFP-CDC48A* expression from the four-cell stage in every cell until the globular embryo stage. At the early heart stage, *pCDC48A:YFP-CDC48A* expression becomes restricted to the protodermal cell layer (Fig. 7a). Similarly, expression of the functional *pWOX2(6.5 kb):GFP-WOX2* reporter (Fig. S6) is detected in all cells of the embryo proper until the early heart stage and is then restricted to the protoderm (Fig. 7b). We confirmed their overlapping expression patterns in embryos co-expressing *pCDC48A:YFP-CDC48A* with a functional *pWOX2(3.5 kb):tdTomato-WOX2* (Fig. 7c, Fig. S5).

To address whether and where the intracellular localization of CDC48A and WOX2 proteins overlap as expected for interacting proteins, we co-transformed *35S:GFP-CDC48A* and *35:tdTomato-WOX2* into *Arabidopsis* leaf mesophyll protoplasts and *Nicotiana benthamiana* leaf cells. We found both proteins co-localized in the nucleus in both species (Fig. 7d–g). In addition, GFP-CDC48A was also present in the cytoplasm in both assays (Fig. 7d–g).

Thus, *pCDC48A:YFP-CDC48A* and *pWOX2(6.5 kb):GFP-WOX2* expression patterns are strikingly similar during early embryogenesis, and their intracellular localizations overlap in the nucleus, consistent with their putative interaction.

### CDC48A does not affect WOX2 protein stability or nuclear accumulation in the embryo

Considering its established canonical function, we then asked how CDC48A might affect WOX2 function. First, a CDC48 function in targeting client proteins toward degradation has been reported in many studies (Begue et al. 2017). However, we found that the levels of WOX2-YFP-GR protein were indistinguishable among *cdc48a-1*/+, CDC48A overexpressor, and wild-type roots (Fig. S7), indicating that CDC48A is not required for ectopically-expressed WOX2’s stability. Second, since previous studies also reported a role of CDC48 in the nuclear localization of client proteins (Ndoja et al. 2014; Wilcox and Laney 2009), we considered whether CDC48A affects the subcellular localization of WOX2. To this end, we compared the subcellular distributions of *gWOX2-GFP* between wild-type and segregating *cdc48a-1* embryos. Because the first protoderm developmental defect occurs at the 16-cell stage, we focused on the 8-cell, 16-cell, and early globular stages. In all three developmental stages, we observed neither a change in nuclear to cytoplasmic GFP signal ratio nor a change in total GFP content in both the nucleus and the cytoplasm between the wild-type and *cdc48a-1*/+ backgrounds (Fig. S8a–c). These results demonstrate that CDC48A is not a limiting factor for the nuclear localization and stability of the WOX2 protein in the embryo.

## Discussion

WOX2 is a central regulator for protoderm and shoot meristem initiation during early embryo patterning (Zhang et al. 2017). However, the molecular mechanisms of its function have remained enigmatic. Here, we isolated CDC48A as a putative interactor of WOX2 and showed that it is required for protoderm and shoot meristem initiation similar to WOX2. In the following, we will address the role of CDC48A and its implications for WOX2 function in embryo patterning.

### WOX2 expression in the root results in shoot-like reprogramming

Since the cells expressing WOX2 in the embryo are too scarce, we have chosen the overexpression of WOX2 in the root as a proxy for its function in shoot meristem initiation to search for interactors by IP–MS/MS. Several observations support this choice: WOX2 overexpression in the root induced the formation of shoot meristem-like structures and increased expression of two WOX2 downstream genes, the stem cell marker gene *CLV3* and *PHB*, which mediates WOX2 function in embryonic shoot meristem initiation (Zhang et al. 2017). A similar conversion of root to shoot cell fates has been previously reported for the *WUS* gene (Gallois et al. 2004; Negin et al. 2017), the major regulator of stem cell maintenance. Furthermore, WOX2 can replace WUS in postembryonic shoot meristem maintenance, supporting similar protein functions (Dolzblasz et al. 2016). Although broadly overexpressed in our experiment, the discrete appearance of proliferating bulges rather than a complete conversion of all root cells and the relatively modest increase of *CLV3* and *PHB* expression suggest that WOX2 induces stable shoot meristem features only at restricted foci. A plausible interpretation is that initial differences between cells and/or confinement of cell fates by lateral inhibition limit the formation of novel structures to a small region within the *WOX2*-expressing cells. Similar observations have been made for the induction of ectopic shoot meristem when ubiquitously expressing *WUS* (Lu et al. 2023; Zuo et al. 2002).

### WOX2 and CDC48A are required for protoderm and shoot meristem initiation

The expression patterns of WOX2 and CDC48A proteins overlap during early embryo patterning, *cdc48a-1*/+, and *wox2-2* embryos display strikingly similar defects in protoderm division patterns, and *cdc48a-1*/+ *wox2-2* double mutants display non-additive effects at this stage. Together with the delayed expression onset of the shoot meristem stem cell marker *CLV3* during embryogenesis and a lack of a functional shoot meristem at the seedling stage, these findings suggest that WOX2 and CDC48A act together during protoderm formation and shoot meristem initiation.

That an extra copy of WOX2 compensates for the absence of CDC48A activity, specifically in protoderm formation, is curious. One plausible explanation is that CDC48A potentiates physiological levels of WOX2 function in the wild type, the absence of which can be overcome by a surplus of WOX2 protein. A role of CDC48A in regulating *Arabidopsis* cell divisions is also suggested by previous reports. Park et al. (2008) found that *cdc48a-1* primary roots post-germination exhibited abnormally orientated cell division planes in the meristematic region (Park et al. 2008). Furthermore, during cytokinesis in *Arabidopsis*, CDC48A has been shown to interact with the member of the SNARE protein complex, the syntaxin 5 ortholog SYP31, possibly mediating membrane fusion and/or disassembly at the cell division plane (Rancour et al. 2002). Finally, *Arabidopsis* CDC48A has been found to promote mitotic spindle assembly and cell division in yeast cells (Feiler et al. 1995). While these findings support a role of CDC48 during cytokinesis, understanding how its interaction with WOX2 influences protodermal cell division patterning requires further studies.

One obvious question is whether the defects in shoot meristem stem cell initiation of *wox2* and *cdc48a-1* mutants resulted from the previous failure to separate the protodermal layer correctly or whether these events reflect separate functions of WOX2/CDC48A. Whereas the severe pleiotropic defects make addressing this question in the *cdc48a-1* mutant difficult, two observations argue against this possibility. First, our previous analysis showed that protodermal defects in genetic combinations of the *wox2* mutant are more frequent than shoot meristem defects (Breuninger et al. 2008). Furthermore, we report that an extra *WOX2* copy can complement the protodermal of the *cdc48a-1* mutant but not its shoot meristem defects. What could the downstream processes be? While the targets of WOX2 in protoderm formation have yet to be revealed, it is tempting to speculate that the WOX2/CDC48A interaction may mediate the downregulation of *MIR166B* expression in the apical protoderm and the subsequent activation of *HD-ZIP III* transcription factors and, ultimately, the cytokinin to auxin ratio during shoot stem cell initiation, as reported for *WOX2* (Zhang et al. 2017). Since the pleiotropic effects of *cdc48a-1/*+ seedlings hamper a more detailed comparison with the *wox2* or *wox1235* seedlings, further work is necessary to unveil the molecular mechanisms of WOX2/CDC48A function.

How common is the function of CDC48A in protoderm formation and shoot meristem initiation in the plant kingdom? In *Nicotiana glutinosa*, the NgCDC48 homolog was found to be crucial for normal vegetative and reproductive shoot development, with its loss of function resulting in seedling lethality (Bae et al. 2009). However, the underlying mechanisms and a potential connection to a WOX2 homolog in this species are unknown. Functional studies of CDC48 homologs in other plant species are currently lacking.

### WOX2 and CDC48A proteins interact

We found that WOX2 and CDC48A proteins interact by different experiments: IP–MS/MS, BiFC in transiently transformed *Arabidopsis* cells, and Co-IP in stable *Arabidopsis* lines. We tested two hypotheses for well-known CDC48A functions on WOX2: protein degradation and nuclear localization. First, many studies show that CDC48A can segregate client proteins toward proteasomal degradation, for example, by promoting centromere disassembly during the cell cycle (Merai et al. 2014). However, our results suggest that this is unlikely for WOX2. *wox2-2* (and the enhanced *wox1235* quadruple mutant) and *cdc48a-1*/+ display similar defects in protoderm and shoot meristem development, not opposite ones as one would expect if CDC48A promoted WOX2 proteolysis. Nevertheless, it is also conceivable that CDC48A promotes WOX2 function by removing misfolded WOX2 proteins and submitting it to proteolysis, as has been shown for the CDC48A client protein SOMATIC EMBRYOGENESIS RECEPTOR KINASE1 (Aker and de Vries 2008). However, we have not detected any effect of *cdc48a-1*/+ or CDC48A-overexpressing plants on the signal strength of GFP-tagged WOX2 protein, making a proteolytic-based mechanism unlikely. Second, it has also been reported that CDC48 can mediate the nuclear localization of client transcription factors (Ndoja et al. 2014; Wilcox and Laney 2009). However, we did not detect any changes in the nuclei/cytoplasmic ratio of GFP-tagged WOX2 protein in embryo cells, arguing against such a mechanism during embryo patterning. Further studies on the WOX2–CDC48A interaction are required to pinpoint its precise mechanism influencing embryo patterning.

## Materials and methods

### Plant materials and growth conditions

The *Arabidopsis thaliana* accession used in this study was Columbia (Col-0). *wox2-2* is a T-DNA insertion line in the Col-0 background described before (Haecker et al. 2004). *wox1-2 wox2-4 wox3-2 wox5-1* (*wox1235*) all have T-DNA insertions in the Col-0 background and were described before (Zhang et al. 2017). *cdc48a-1*, *cdc48a-2,* and *cdc48a-3* are T-DNA insertion mutants in the Col-0 background previously described (Park et al. 2008). *pCDC48A:YFP-CDC48A cdc48a-1/*+ was also obtained from Dr. Sookhee Park (Park et al. 2008). The mutants, transgenic lines, plasmids, primers, and genotyping methods used in this study are summarized in Tables S8, S9, S10, S11, and S12.

*Arabidopsis* and tobacco (*Nicotiana benthamiana*) seeds were sown on wet soil in pots, stratified at 4 °C for 2 days, and then grown in a phytochamber at 21 °C under long-day conditions (16 h light, 8 h dark). For seeds sown on ½ Murashige and Skoog (MS) plates, seeds were sterilized with 70% ethanol for 1 min and then with 1.2% NaClO for 10 min, followed by a 3-time wash with autoclaved distilled water. Seeds were then sown on ½MS plates, stratified at 4 °C for 2 days, and allowed to grow in a growth chamber (Percival Scientific) at 22 °C under long-day conditions (16 h light, 8 h dark). ½ MS media contained 2.15 g/L Murashige and Skoog Medium (Duchefa), 0.5 g/L MES hydrate with pH of 5.7, adjusted by KOH and 8-10 g/L agar (Plant Agar, Duchefa).

### Plasmid construction and plant transformation

DNA fragments were amplified from *Arabidopsis* genomic DNA by PCR with specific primers (Table S11) by Phusion DNA polymerase (NEB) according to the manufacturer’s instructions. PCR fragments were ligated into the pJET1.2 cloning vector (Thermo Fisher Scientific Inc.) following the manufacturer’s instructions. Ligations of DNA fragments into pGreenII binary vectors following specific restriction enzyme digestions were done using T4 DNA Ligase (NEB). Up to 5 µl ligation products were transformed into 50 µl *E.coli* XL1 Blue electrocompetent cells. Colony PCRs and DNA sequencing were done to confirm positive clones.

To create *pRPS5A:WOX2-YFP-GR*, WOX2 CDS was amplified with BamHI flanking sites with primers ERp008/009, and vYFP was amplified with BamHI site GAGA linker-NcoI sites using primers ERp045/046. pRPS5A (Weijers et al. 2001), WOX2 CDS and vYFP were then digested with BamHI digestion and ligated into a pGreenII vector with a rat glucocorticoid receptor (GR) domain, a NOS terminator, and kanamycin resistance for both bacteria and plant.

To create *gWOX2-GFP*, 6.5 kb upstream, the transcription start site of *WOX2* as the promoter was amplified with AscI and HindIII flanking sites by primers oWG164/165. GFP was amplified from *pWOX2:H2B-GFP* (Zhang et al. 2017) and flanked by HindIII and XmaI-linker using primers oWG168/169. WOX2 open reading frame and 1.7 kb 3ʹUTR were amplified, and XmaI was flanked at both sides by primers oWG166/167. PCR fragments in pJET1.2 were digested with the indicated restriction enzymes and ligated into a pGreen II vector with kanamycin and norflurazon resistance for bacteria and plants, respectively.

For *35S:CDC48A-FLAG*, CDC48A CDS with three FLAG tags was amplified with BamHI and NotI flanking sites by primers oWG216/217. PCR fragments in pJET1.2 were digested with the indicated restriction enzymes and ligated into a pGreenII vector carrying a 35S promoter and a 35S terminator before and after the insertion site and kanamycin and norflurazon resistance for bacteria and plant, respectively.

To create *pWOX2:tdTomato-WOX2*, 3.5 kb upstream, the transcription start site of *WOX2* was amplified from *pWOX2:H2B-GFP* (Zhang et al. 2017) and cloned into a pGreenII vector carrying a NOS terminator by ligation independent cloning (LIC) (De Rybel et al. 2011). The adaptor sequence is GATCCCTAGTTGGAATTGGTTTAAACCCAACTCCATAAGG, containing a PmeI site. The vector was then linearized by SpeI and SalI. tdTomato-GAGA linker was amplified by primers oWG470/471 and WOX2 CDS was amplified by primers oWG472/473. Both PCR fragments were then amplified again into tdTomato-GAGA linker-WOX2 by Gibson assembly (Gibson et al. 2009) and ligated into a pGreenII vector, which has kanamycin and MTX resistance for bacteria and plant, respectively.

For the BiFC assays, the CDS of *WOX2* was amplified by primers oWG90/91 with added EcoRI and SacII restriction sites and ligated into pRT-SPYNE, a vector with *35S:nYFP* (Walter et al. 2004). The CDS of CDC48A was amplified by primers oWG94/95 and ligated into pRT-SPYCE (Walter et al. 2004), a vector carrying *35S:cYFP*. CDC48A ΔN was amplified by oWG186/ oWG95. CDC48A ΔC was amplified by oWG94/ oWG188. CDC48A ΔC + D2 was amplified by oWG94/ oWG190. SacII restriction sites at both ends flanked all mutated CDC48A variants. PCR fragments in the pJET1.2 vector were first digested by SacII and ligated into SacII-linearized pRT-SPYCE with *35S:cYFP*.

For the WOX2–CDC48A co-localization assays, *35S:NTF*, *35S:tdTomato-WOX2*, *35S:GFP-CDC48A,* and *35S:tdTomato-CDC48A* were generated by assembling the linear fragments following the AQUA cloning procedure (Beyer et al. 2015). For the first three constructs, NTF, tdTomato coding sequence, WOX2 CDS, GFP coding sequence and CDC48A CDS were amplified by primer pairs oWG454/ oWG455, oWG440/ oWG441, oWG442/ oWG443, oWG448/ oWG449, oWG450/ oWG451. tdTomato coding sequence was amplified from pWOX2:LTI6b-tdTomato (Zhang et al. 2017). For *35S:tdTomato-CDC48A*, tdTomato coding sequence, and CDC48A CDS were amplified by primers oWG440/452 and oWG453/ oWG451, respectively. After the AQUA cloning procedure, PCR fragments were purified and cloned into linearized vector pRT-SPYNE by NotI/ XbaI digestion (Beyer et al. 2015).

The plasmids generated in this study are listed in Table S10. The binary vectors with the corresponding constructs were first transformed into *Agrobacterium tumefaciens* strain GV3101 with the pSOUP helper plasmid (Koncz and Schell 1986). Plant transformations in *Arabidopsis thaliana* were carried out using the floral dip method (Clough and Bent 1998).

### Dexamethasone treatment of roots

Plants expressing *pRPS5A:WOX2-YFP-GR* were grown on ½MS medium for 4 days and then transferred to ½MS supplemented with either 10 μM dexamethasone in H_2_0 (Sigma-Aldrich®, D1756) or 10 μM ethanol in H_2_0 (mock treatment). Seedlings were allowed to grow on the respective agar for a selected amount of time for further analyses. The same treatment was done on control wild-type seeds, where needed.

### Gene expression analyses

For RNA extraction, around 100 mg of root tips from 6-day-old *pRPS5A:WOX2-YFP-GR* seedlings following a 48-h + DEX/mock treatment were taken from one sample and frozen immediately in liquid nitrogen. RNeasy Plant Mini Kit (Qiagen) was used to isolate total mRNA according to the manufacturer’s instructions. Gene expression analyses were determined by qRT-PCR. Reverse mRNA transcription was performed from 1 µg total RNA using Superscript III First-Strand Synthesis Super Mix for qRT-PCR (Invitrogen) following the manufacturer’s instructions. The qRT-PCR reaction was performed using LightCycler 480 SYBR Green I Master (Roche) and a LightCycler 480 (Roche) under the following conditions: 98 °C for 5 min, 40 cycles of 98 °C for 10 s, 60 °C for 20 s, and a final 30-s extension at 72 °C in the cycling stage, 95 °C for 5 s, 60 °C for 20 s, and 97 °C for 1 min in the melt curve stage. Data were analyzed with LightCycler 480 software version 1.5.0.39 (Roche). *TIP41* (AT4G34270) was used as an internal reference for qRT-PCR as its expression was experimentally reported to be sufficiently stable for seedlings grown on ½MS with or without DEX (Han et al. 2013). The qRT-PCR primers used in this study are listed in Table S11. The relative expression level of each gene is presented as the normalized RQ, as previously described (Rieu and Powers 2009).

### Immunoprecipitation followed by tandem mass spectrometry (IP–MS/MS)

2.5 g of fresh weight root material from 5-day-old *pRPS5A:WOX2-YFP-GR* seedlings after a 4-h 10 μM Dexamethasone (DEX; Sigma-Aldrich®, D1756) treatment, performed as described above, were cut off from the base of the hypocotyl. Harvested roots were then used for immunoprecipitation and tandem mass spectrometry, and follow-up statistical analysis was done as previously reported (Wendrich et al. 2017). Enriched proteins were normalized to *35S:YFP* roots following the same DEX treatment. Each transgenic line had 3 biological replicates. *Arabidopsis* TAIR10 database was used for data search.

### Protoplast isolation and transfection for BiFC and co-localization analyses

For the bimolecular fluorescence complementation (BiFC) assays, *Arabidopsis* leaf mesophyll protoplasts were isolated and transfected for transient gene expression, as previously reported (Wu et al. 2009; Yoo et al. 2007). After overnight incubation in darkness following transfection with respective plasmids (Table S10), fluorescence signals from protoplasts were observed, and frequencies of observations were counted under a Zeiss Axio Imager microscope. Representative images were taken by a Zeiss LSM700 confocal laser scanning microscope.

### Tobacco leaf infiltration for co-localization analyses

The respective plasmids (Table S10) were transformed into *Agrobacterium tumefaciens* strain GV3101 for transient expression in *Nicotiana benthamiana* leaves. The infiltration of tobacco leaves was done as previously described (Sparkes et al. 2006). 2 days following infiltration, gene expression via reporter fluorescence signal was confirmed under the Zeiss Axio Imager microscope, and images were taken by a Zeiss LSM700 confocal laser scanning microscope.

### Immunoprecipitation assays

For both co-immunoprecipitation (Co-IP) and immunoprecipitation assays, *Arabidopsis* stable lines indicated in the respective figures and Table S9 were used. *pRPS5A:WOX2-YFP-GR/35S:CDC48A-FLAG* is the result of a cross between *pRPS5A:WOX2-YFP-GR* and *35S:CDC48A-FLAG*. About 1 g of 6-day-old seedlings per transgenic line was ground in liquid nitrogen with a mortar and pestle. 1 ml cold lysis buffer was added to the mortar and ground thoroughly after it was thawed. The protocol provided by µMACS™ Epitope Tag Protein Isolation Kit (Miltenyi Biotec) was followed afterward. Before adding anti-GFP, anti-FLAG beads, or anti-Tubulin beads, 50 µl lysate was taken as the input. For western blots, the inputs and immunoprecipitated samples were loaded on 8–12% PAGE gels, depending on the protein size. Then, the blots were probed with anti-GFP (ab290), anti-FLAG (Sigma), or anti-tubulin (Sigma) antibodies, each with a 1:5000 dilution.

### Nomarski microscopy

For Nomarski microscopy (or DIC microscopy) analyses, siliques were first dissected on a double-sided sticky tape and cut open along the replum under a stereomicroscope using a fine needle. Samples were cleared in chloral hydrate solution (80 g chloral hydrate, 30 g H_2_O, and 10 g glycerol dissolved for 24 h with gentle stirring at 4 °C) on a microscope slide for 2–20 h at room temperature, depending on the embryo stage to be observed. Cleared samples were then observed and imaged under a Zeiss Axioscope2 using the 40× objective.

#### Confocal laser scanning microscopy

Developing seeds were dissected from siliques under a stereomicroscope and then mounted on microscope slides with 10% glycerol. Seeds were gently squeezed under the coverslip to extract embryos. Fluorescence was visualized with a Zeiss LSM700 confocal laser scanning microscope under the 40× objective, and images were captured with the ZEN 2010 software program. FM4-64 counterstaining of embryos was done as previously described (Rigal et al. 2015). For observing *pRPS5A:WOX2-YFP-GR* in the root, root tips cut off from seedlings were mounted in 50 µM propidium iodide (PI) and left to incubate at room temperature for 2 min. The 10× objective was used to observe whole root tips, and the 40× objective was used to discern and image reporter subcellular localization. The settings for excitation/emission were 448 nm/550–570 nm (YFP/GFP) and 550 nm/ > 610 nm (tdTomato/mCherry/PI/FM4-64). For multi-channel imaging, each channel was captured simultaneously.

#### Quantification and statistical analyses

Pairwise comparisons for categorical data were done by Fisher’s exact tests. *p*-values less than 0.05 were considered statistically significant. Bonferroni correction was applied for multiple comparisons. Mean and integrated signal intensities were quantified with ImageJ version 1.53t (imagej.net) from a single focal plane for fluorescence intensities. Quantitative data were analyzed by the Student’s *t* test or ANOVA with the relevant Post Hoc test for multiple comparisons using GraphPad Prism version 9.4 (GraphPad Software, Inc.). All data were plotted using GraphPad Prism version 9.4. The statistical tests used for each experiment are labeled in the relevant figure captions.

### Supplementary Information

Below is the link to the electronic supplementary material.

Supplementary file1 (DOCX 1593 KB)

## Data Availability

All the data in this study are included in the figures and tables.
