# Supplementary material for: CDC48A, an interactor of WOX2, is required for embryonic patterning in Arabidopsis thaliana
Source: Plant Cell Rep. 2024 Jun 15;43(7):174. doi: 10.1007/s00299-024-03158-2 (PMC11180018; doi:10.1007/s00299-024-03158-2)
Supplement: Supplementary file 1 — Supplementary file1 (DOCX 1593 KB) [file 299_2024_3158_MOESM1_ESM.docx]

**Table S1.** **qRT-PCR Cp values for *CLV3* and *PHB* from *pRPS5A:WOX2-YFP-GR* and wild-type (WT) 6-day-old roots.**

| Sample | Cp (*CLV3*) | Cp (*PHB*) | Cp (*TIP41*) |
| --- | --- | --- | --- |
| WT mock 1 | 36.04 | 27.52 | 25.22 |
| WT mock 2 | 36.28 | 27.55 | 25.19 |
| WT mock 3 | 38.96 | 27.64 | 25.11 |
| WT DEX 1 | 36.07 | 27.72 | 25.14 |
| WT DEX 2 | 36.39 | 27.48 | 24.94 |
| WT DEX 3 | 36.00 | 27.52 | 25.22 |
| *pRPS5A:WOX2-YFP-GR* mock 1 | 35.62 | 26.83 | 24.52 |
| *pRPS5A:WOX2-YFP-GR* mock 2 | 36.05 | 26.70 | 24.54 |
| *pRPS5A:WOX2-YFP-GR* mock 3 | 36.39 | 26.81 | 24.55 |
| *pRPS5A:WOX2-YFP-GR* DEX 1 | 32.80 | 24.56 | 23.80 |
| *pRPS5A:WOX2-YFP-GR* DEX 2 | 32.97 | 24.26 | 23.59 |
| *pRPS5A:WOX2-YFP-GR* DEX 3 | 32.66 | 24.18 | 23.72 |
| 4-day-old roots were incubated with either 10μM Dexamethasone (DEX) or mock solution for 2 days. *TIP41* (AT4G34270) was used as the reference gene. Data shows three biological repeats for each treatment. WT, wild type | | | |

**Table S2. List of enriched proteins from the IP-MS/MS assay.**

| Ratio | *p*-value | Protein name | Gene name |
| --- | --- | --- | --- |
| 2186.12 | 0.0128 | WUSCHEL-related homeobox 2 | WOX2 |
| 498.199 | 5.7E-05 | Putative membrane protein ycf1 | YCF1-A |
| 468.791 | 4.5E-06 | Mitochondrial outer membrane protein porin 1 | VDAC1 |
| 404.035 | 7.2E-08 | S-adenosylmethionine synthase 4 | METK4 |
| 272.033 | 1.7E-06 | 40S ribosomal protein Sa-1 | RPSaA;P40 |
| 164.346 | 4.4E-06 | GTP-binding nuclear protein Ran-1;2;3 | RAN1;RAN2;RAN3 |
| 146.386 | 6.9E-06 | Protein TOPLESS | TPL |
| 145.899 | 3.5E-06 | Heat shock 70 kDa protein 3 | HSP70-3 |
| 123.371 | 0.04928 | LOS1 | LOS1 |
| 122.4 | 0.03881 | Heat shock protein 90-4;3;2 | HSP90-4;HSP90-3;HSP90-2 |
| 99.6734 | 4.1E-05 | ATP synthase subunit beta-3 |  |
| 98.156 | 4.1E-08 | S-adenosylmethionine synthase 3 | METK3 |
| 48.1201 | 5.2E-07 | Heat shock 70 kDa protein 9, mitochondrial | HSP70-9 |
| 28.0586 | 3.5E-06 | 14-3-3-like protein GF14 chi | GRF1 |
| 19.3823 | 0.0012 | Cell division control protein 48 homolog A | CDC48A |
| 16.6101 | 0.00018 | Heat shock 70 kDa protein 1 | HSP70-1;HSC70-1 |
| 10.781 | 0.02179 | Histone H4 |  |
| 6.86477 | 0.00048 | 40S ribosomal protein S2-3 | RPS2C |
| 5-day-old seedlings of *pRPS5A:WOX2-YFP-GR* were treated with 10μM DEX for 4h, and proteins were extracted from the roots for the IP-MS/MS assay. *35S:YFP* roots under the same treatment were used as a control. Ratio is the log10 value of the enrichment.  *p*-value is calculated by *t*-test from three biological replicates. | | | |


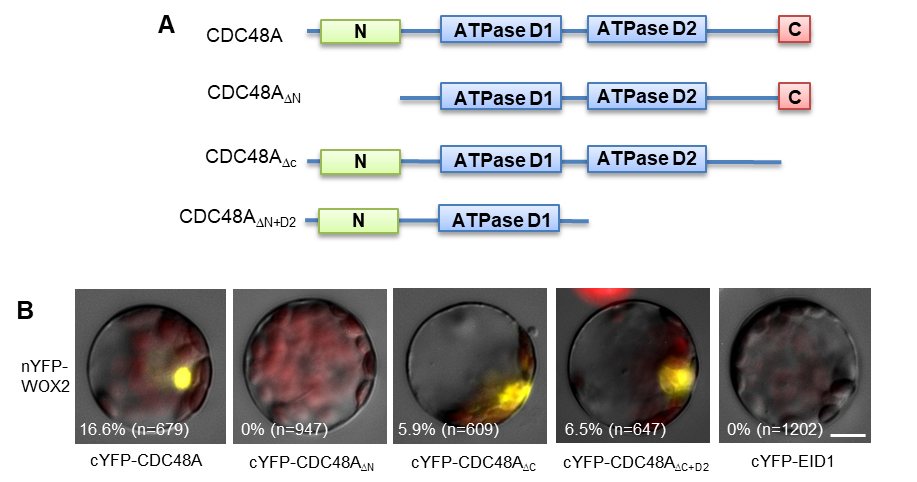


**Figure S1. CDC48A interacts with WOX2 through its N terminus.**

**(A)** Schematic representation of CDC48A protein structure with its conserved domains. N, N-terminal domain; C, C-terminal domain.

**(B)** BiFC assay showing interaction between WOX2 and CDC48A variants. EID1 was used as a negative control. The percentages of protoplasts with YFP signal and the total numbers of protoplasts observed are indicated. Scale bar: 20μm.


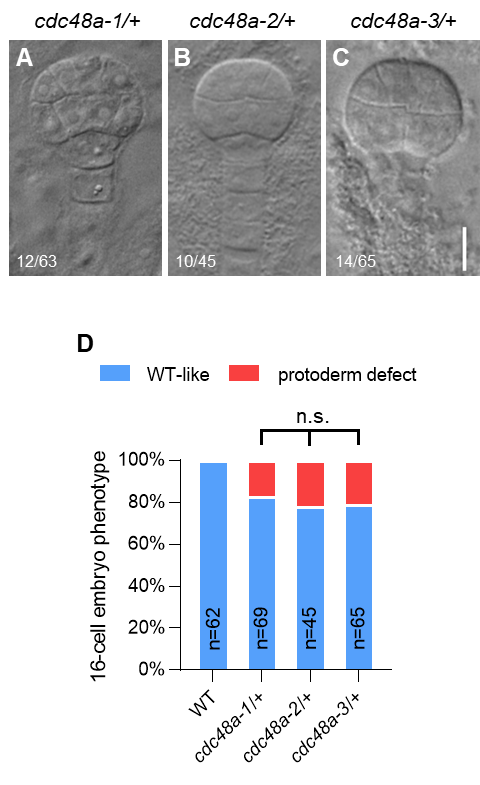


**Figure S2. All three *cdc48a* mutations exhibit similar defects in protoderm formation in 16-cell stage embryos.**

**(A-C)** 16-cell stage embryos from *cdc48a-1*/+, *cdc48a-2*/+, and *cdc48a-3*/+ mother plants showing abnormal anticlinal cell divisions in the apical tier. Scale bar: 10μm

**(D)** Frequencies of 16-cell embryo phenotypes of the indicated genotypes.

WT, wild type. n.s., not significant (Fisher‘s exact test with Bonferroni correction).


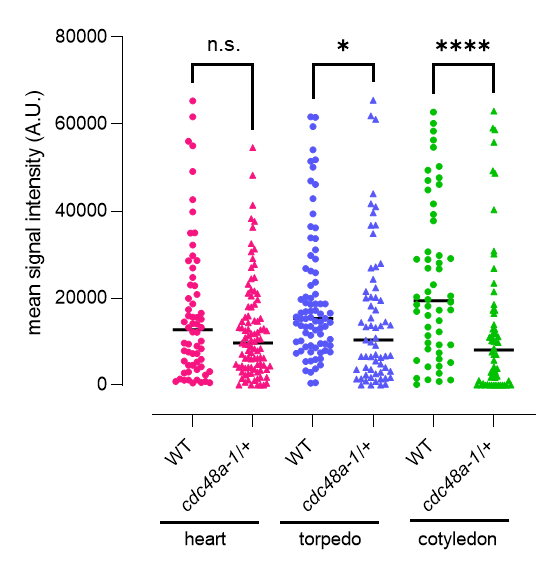


**Figure S3. CDC48A is necessary for the timely initiation and maintenance of *CLV3* expression in developing embryos.**

Mean fluorescence intensities of *pCLV3:er-tdTomato* reporter expression at the heart, torpedo, and bent cotyledon stages from mother plants of the wild type (WT) and *cdc48a-1*/+. Black bars within the data points represent the median. n.s., not significant, *, *p* < 0.05, ***, *p* < 0.001 (Kruskal-Wallis with Dunn’s post hoc test).

**Table S3. Genotypes of the progeny from a *cdc48a-1*/+ mother plant.**

| Genotype | Percentage | Counts |
| --- | --- | --- |
| *cdc48a-1*+/+ | 32.0 | 25 |
| *cdc48a-1* +/- | 50.0 | 39 |
| *cdc48a-1* -/- | 10.3 | 8 |
| not germinated | 7.7 | 6 |
| All seeds (n=78) from two siliques harvested from a *cdc48a-1*/+ mother plant were sown on ½MS plates. 10-day-old seedlings were genotyped by PCR, except for non-germinated ones. | | |

**Table S4. Genetic linkage between *wox5-1* and *cdc48a-1*/+.**

| Genotypes | Expected distribution  in % | Observed distribution  in % |
| --- | --- | --- |
| *wox5-1/+ cdc48a-1*/+  *wox5-1 cdc48a-1*/+  *wox5-1*/+  *cdc48a-1*/+  *wox5-1*  wild type | 33.3  16.7  16.7  16.7  8.3  8.3 | 40.7  0  59.3  0  0  0 |
| Genotype distributions of *wox5-1* and *cdc48a-1* alleles in F2 seedlings of a *wox13 wox2/+ wox5/+ cdc48a/+* F1 mother plant.  Seeds were sown on ½MS and then transferred to soil. Plants were PCR-genotyped four weeks after germination. n=144 | | |

**Table S5. 10-day-old seedling phenotype frequencies (%) of wild type, *cdc48a-1*/+, *wox2-2*, *wox2-2 cdc48a-1*/+, *gWOX2-GFP* and *gWOX2-GFP* *cdc48a-1*/+ plants.**

| Genotype | WT-like | 1DAG-like,  no SAM | fused cotyledons | single cotyledon | not germinated | n |
| --- | --- | --- | --- | --- | --- | --- |
| wild type | 98.9 | 0 | 0 | 0 | 1.1 | 438 |
| *cdc48a-1*/+ | 86.2 | 2.4 | 0 | 0 | 11.7 | 291 |
| *wox2-2* | 90.9 | 0 | 2.3 | 2.3 | 4.5 | 219 |
| *wox2-2 cdc48a-1*/+ | 87.3 | 3.7 | 0.7 | 1.0 | 7.3 | 411 |
| *gWOX2-GFP* | 97.9 | 0 | 0 | 0 | 2.1 | 571 |
| *gWOX2-GFP* *cdc48a-1*/+ | 85.2 | 4.1 | 0 | 0 | 10.7 | 291 |
| All seeds were sown on ½MS plates.  WT-like, wild-type-like; DAG, days after germination. | | | | | | |


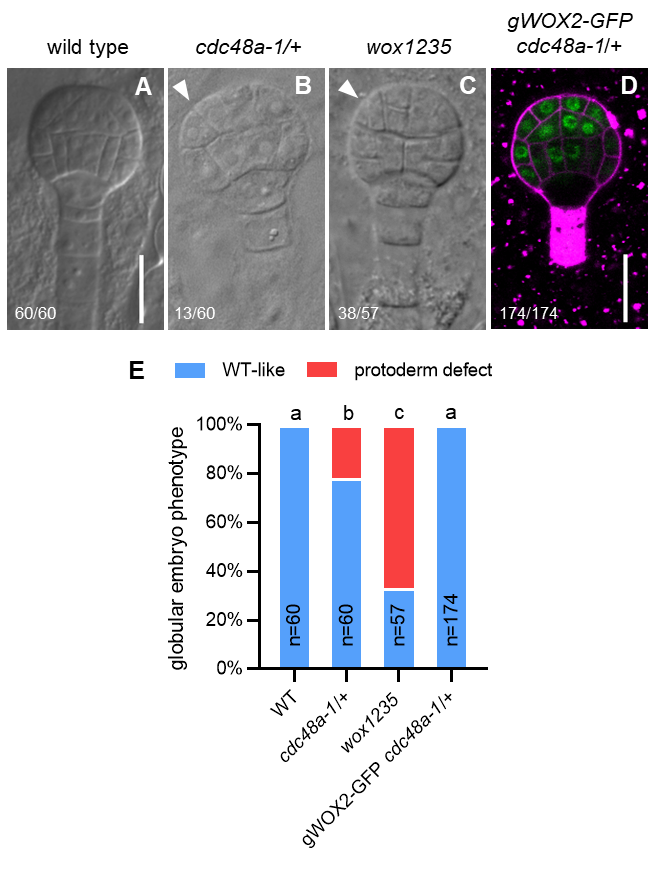


**Figure S4. An extra *WOX2* gene copy rescues the protoderm defect at the globular stage in *cdc48a-1*/+.**

**(A-D)** Early globular embryos from the mother plants of indicated genotypes. White arrowheads indicate aberrant cell division planes. *gWOX2-GFP* transgene contains a genomic version of *WOX2* with a 6.5kb portion upstream of its transcription start site. FM4-64 counterstaining of the plasma membranes is shown (magenta). Scale bars: 20μm.

**(E)** Frequencies of early globular embryo phenotypes observed. a, b and c indicate significance categories (*p* < 0.01) by Fisher’s exact test with Bonferroni correction. WT, wild type.

**Table S6. Developing seed and embryonic developmental stage observations.**

|  | Embryonic stages in cleared ovules  (%) | | | | | Seed morphology (%) | | n |
| --- | --- | --- | --- | --- | --- | --- | --- | --- |
| Genotype | bent cotyledon | torpedo | heart | transition | globular | green seeds | pale seeds |  |
|  | | | | | | | | |
| 6^th^ silique | | | | | | | | |
| wild type | 0 | 43.5 | 29.3 | 27.2 | 0 | - | - | 147 |
| *cdc48a-1*/+ | 0 | 0.5 | 42.4 | 26.1 | 31 | - | - | 184 |
|  | | | | | | | | |
| 10^th^ silique | | | | | | | | |
| wild type | 9.3 | 69.8 | 18.5 | 1.2 | 1.2 | - | - | 172 |
| *cdc48a-1*/+ | 0 | 64.5 | 34 | 1 | 0.5 | - | - | 200 |
|  | | | | | | | | |
| 15^th^ silique | | | | | | | | |
| wild type | 100 | 0 | 0 | 0 | 0 | 100 | 0 | 307 |
| *cdc48a-1*/+ | 89.3 | 8 | 2.7 | 0 | 0 | 92.7 | 7.3 | 150 |
| n, number of seeds. | | | | | | | | |

**Table S7. F1 progeny genotypic ratios from reciprocal crosses between wild-type and *cdc48a-1*/+ plants.**

| Parent (♀ x ♂) | Observed ratio (WT : *cdc48a-1*/+ : *cdc48a-1*) | n |
| --- | --- | --- |
| **♀** WT x ♂ WT | 100% : 0 : 0 | 35 |
| **♀** *cdc48a-1*/+ x ♂ WT | 47.9% : 52.1% : 0 | 94 |
| **♀** WT x ♂ *cdc48a-1*/+ | 98.9% : 1.1% : 0 | 93 |
| All F1 seeds from two siliques were sown on ½MS plates, and were genotyped by PCR at 3 weeks post-germination.  WT, wild type | | |


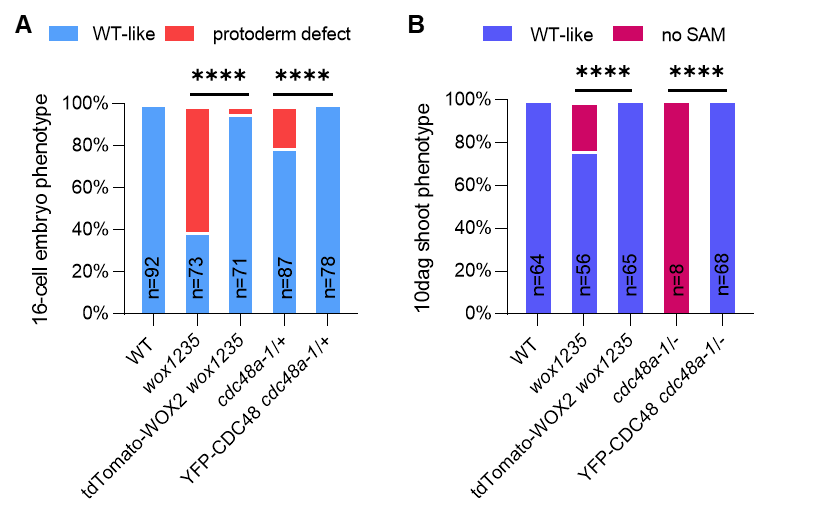


**Figure S5. *pCDC48A:YFP-CDC48A* and *pWOX2(3.5kb):tdTomato-WOX2* are functional.**

**(A)** Frequencies of embryo phenotypes at the 16-cell embryo stage of indicated genotypes. WT, wild type. ****, *p* < 0.0001 (Fisher’s exact test).

**(B)** Frequencies of 10-day-old seedlings with or without a shoot apical meristem (SAM). WT, wild type. ****, *p* < 0.0001 (Fisher’s exact test).


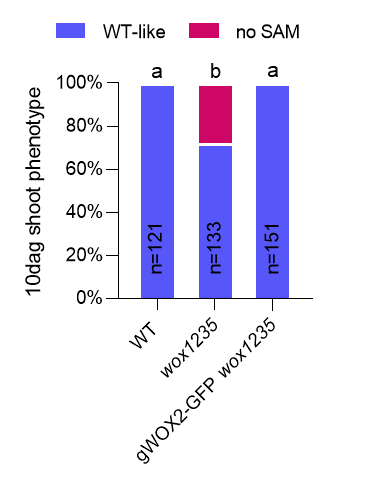


**Figure S6. *pWOX2(6.5kb):GFP-WOX2* (or *gWOX2-GFP)* is functional.**

Frequencies of 10-day-old seedlings from indicated genotypes. *gWOX2-GFP* contains a region 6.5kb upstream of the WOX2 transcription start site and its introns. WT, wild type. a and b indicate significance categories (*p* < 0.00001) by Fisher’s exact test with Bonferroni correction.


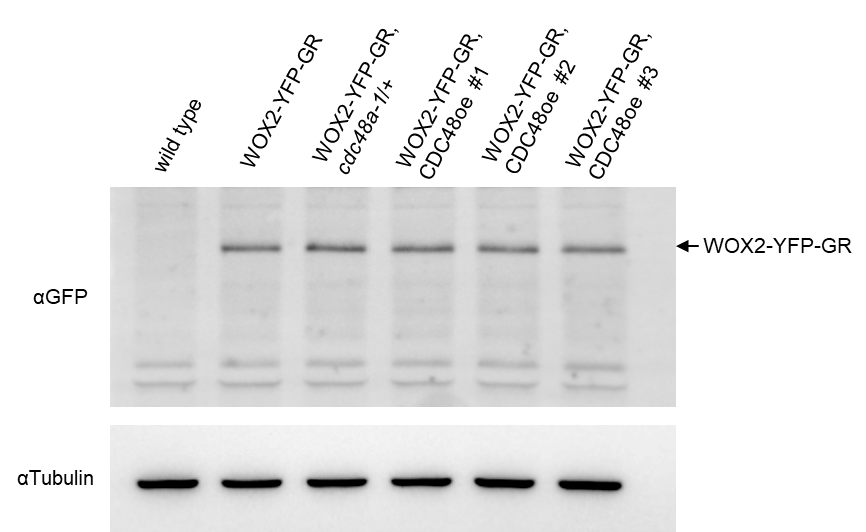


**Figure S7. CDC48A is not required for WOX2 protein stability.**

Western blot of immunoprecipitated proteins from the indicated genotypes. Proteins were extracted from 5-day-old seedlings after 4h of DEX induction, then immunoprecipitated by anti-GFP antibody. Anti-tubulin was used as the loading control.


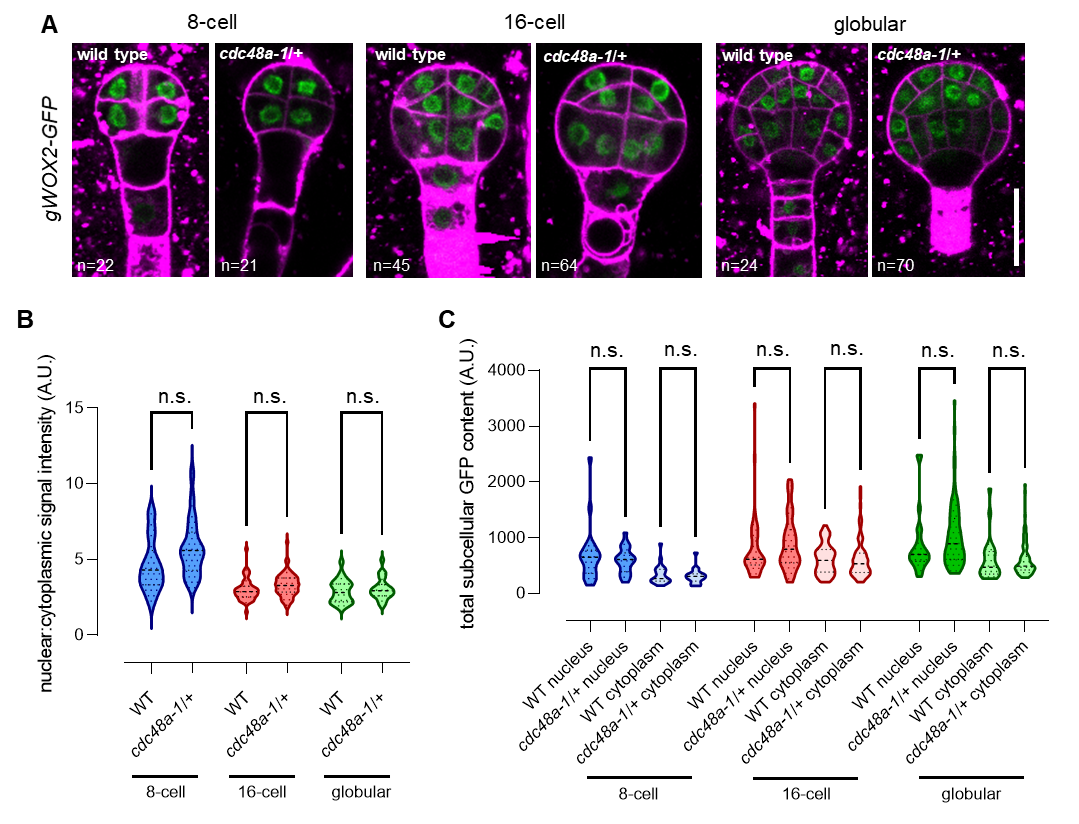


**Figure S8. CDC48A is not required for WOX2’s subcellular localization or stability in embryos.**

**(A)** 8-cell, 16-cell, and early globular stage embryos from wild-type or *cdc48a-1*/+ expressing *gWOX2-GFP* (green). FM4-64 counterstaining of the plasma membranes is shown (magenta). Scale bar: 20μm.

**(B)** Violin plots showing nuclear to cytoplasmic GFP signal intensity ratios between wild-type (WT) or *cdc48a-1*/+ embryos expressing *gWOX2-GFP* at the 8-cell, 16-cell and early globular stages.

**(C)** Violin plots showing integrated GFP signal intensities between wild-type (WT) or *cdc48a-1*/+ 8-cell, 16-cell, and early stage globular embryos expressing *gWOX2-GFP*.

n.s., not significant by One-Way ANOVA with Dunnett‘s T3 post hoc test.

**Table S8. Mutants used in this study.**

| Genotype | Ecotype | Resistance | Mutagenesis type | Origin |
| --- | --- | --- | --- | --- |
| *wox2-2* | Col-0 | Basta | T-DNA | (Haecker, et al. 2004) |
| *wox2-2 cdc48a-1/+* | Col-0 | Basta+Kan | T-DNA | This study |
| *wox1-2wox2-4wox3-2wox5-1* | Col-0 | Kan+Hyg | T-DNA | (Zhang, et al. 2017) |
| *cdc48a-1/+* (SALK_064573) | Col-0 | Kan | T-DNA | (Park, et al. 2008) |
| *cdc48a-2/+* (SALK_116074) | Col-0 | Kan | T-DNA | (Park, et al. 2008) |
| *cdc48a-3/+* (SALK_064893) | Col-0 | Kan | T-DNA | (Park, et al. 2008) |

**Table S9. Transgenic lines used in this study.**

| Clone ID | Transgenic Line | Background |
| --- | --- | --- |
| WG69 | *gWOX2-GFP* | Col-0, *wox1235*, *cdc48a-1****/+*** |
| ZJ03 | *pRPS5A:WOX2-YFP-GR* | Col-0 |
| WG90 | *35S:CDC48A-FLAG* | Col-0*, cdc48a-1/+* |
| ZJ03+WG90 | *pRPS5A:WOX2-YFP-GR/ 35S:CDC48A-FLAG* | Col-0 |
| WG141 | *pWOX2:tdTomato-WOX2* | Col-0*, wox1235* |
| - | *pCDC48A:YFP-CDC48A* | *cdc48a-1* |
| LC68 | *pCLV3:ER-tdTomato* | Col-0*, wox1235, cdc48a-1/+* |
| *pCDC48A:YFP-CDC48A cdc48a-1* was obtained from Park et al., 2008. | | |

**Table S10. Plasmids used in this study.**

| Clone ID | Construct | Resistance (bacteria/plant) | Use | Origin |
| --- | --- | --- | --- | --- |
| WG69 | *gWOX2-GFP* | Kan/Norf | WOX2 reporter | This study |
| ZJ03 | *pRPS5A:WOX2-YFP-GR* | Kan/Kan | IP-MS, Co-IP | This study |
| WG90 | *35S:CDC48A-FLAG* | Kan/Norf | Co-IP | This study |
| WG23 | *35S:nYFP-WOX2* | Amp/Kan | BiFC | This study |
| WG25 | *35S:cYFP-CDC48A* | Amp | BiFC | This study |
| WG80 | *35S:cYFP-CDC48A_ΔN_* | Amp | BiFC | This study |
| WG82 | *35S:cYFP-CDC48A_ΔC_* | Amp | BiFC | This study |
| WG84 | *35S:cYFP-CDC48A_ΔC+D2_* | Amp | BiFC | This study |
| - | *35S:nYFP-ASK1* | Amp | BiFC | (Stolpe, et al. 2005) |
| - | *35S:cYFP-EID1* | Amp | BiFC | (Stolpe, et al. 2005) |
| WG141 | *pWOX2:tdTomato-WOX2* | Kan/MTX | co-localization | This study |
| - | *pCDC48A:YFP-CDC48A* | Spec/Kan | co-localization | (Park, et al. 2008) |
| - | *35S:ER-mCherry* | Kan | co-localization | (Nelson, et al. 2007) |
| WG134 | *35S:tdTomato-WOX2* | Amp | co-localization | This study |
| WG136 | *35S:GFP-CDC48A* | Amp/Kan | co-localization | This study |
| WG137 | *35S:tdTomato-CDC48A* | Amp | co-localization | This study |
| WG138 | *35S:NTF* | Amp | co-localization | This study |
| LC68 | *pCLV3:er-tdTomato* | Kan/MTX | stem cell marker | (Zhang, et al. 2017) |

**Table S11. Primers used in this study**

| Primer ID | Sequence (5' to 3') | Use |
| --- | --- | --- |
| HBp01 | CGAAGTAAACGCAGGAACAGCAAGC | Genotyping |
| HBp02 | AATCAATAAACGGCTGCTCTCGG | Genotyping |
| HBp130 | GGATCCATGTGGACGATGGGTTACAACG | Genotyping |
| TL13 | GCCTTTTCAGAAATGGATAAATAGCCTTGCTTCC | Genotyping |
| TL43 | TGGTTCACGTAGTGGGCCATCG | Genotyping |
| TL63 | AATCTTACTATATATATGGATATATACACAGGCC | Genotyping |
| TL64 | AATCTGATCAGTTGTTGGAGTTCTAAGAC | Genotyping |
| pZJ15 | AGCTGTTGCCCGTCTCACTGGTG | Genotyping |
| pZJ16 | CTAAGTGTTTGGAGATAGCATCAC | Genotyping |
| pZJ17 | ACATGGGAGAAGGATGAGAGCAGC | Genotyping |
| pZJ20 | CGTCAATTTGTTTACACCAC | Genotyping |
| pZJ21 | GATATATACCTCTGGTTGCG | Genotyping |
| oWG191 | ATTTTGCCGATTTCGGAAC | Genotyping |
| oWG192 | ATCAACAGGCAGGATGTGAAC | Genotyping |
| oWG193 | AAAACAATAGGGGTCGGTTTG | Genotyping |
| oWG194 | CCAGCTGAATCTTCAGACTCG | Genotyping |
| oWG195 | ATCTCTGTGTCTGGAGCAACC | Genotyping |
| oWG196 | AAGAAGTCTCCGAACCGTCTC | Genotyping |
| oWG197 | CACGAGCGATCAAAGTCTTTC | Genotyping |
| CLV3 qRT-PCR F | GTTCAAGGACTTTCCAACCGCAAGATGAT | qRT-PCR |
| CLV3 qRT-PCR R | CCTTCTCTGCTTCTCCATTTGCTCCAACC | qRT-PCR |
| PHB qRT-PCR F | GCTAACAACCCAGCAGGACTCCT | qRT-PCR |
| PHB qRT-PCR R | TAAGCTCGATCGTCCCACCGTT | qRT-PCR |
| TIP41 qRT-PCR F | GTGAAAACTGTTGGAGAGAAGCAA | qRT-PCR |
| TIP41 qRT-PCR R | TCAACTGGATACCCTTTCGCA | qRT-PCR |
| ERp008 | GGTAATGGGTTGGATCCAAGGTTTAATG | plasmid construction |
| ERp009 | CATTAAACCTTGGATCCAACCCATTACC | plasmid construction |
| ER045 | GATCCAGGAGCAGGAGCAGGAGCAGGAGC | plasmid construction |
| ER046 | CAGGAGCAGGAGCAGGAGCAGGAGCCATG | plasmid construction |
| oWG90 | TTGAATTCATATGGAAAACGAAGTAAACGCAG | plasmid construction |
| oWG91 | TTCCGCGGTTACAACCCATTACCATTAC | plasmid construction |
| oWG94 | TTCCGCGGAATGTCTACCCCAGCTGAATC | plasmid construction |
| oWG95 | TTCCGCGGCTAATTGTAGAGATCATCAT | plasmid construction |
| oWG164 | TTGGCGCGCCCTTTCTCGTAACCCAAATTCTC | plasmid construction |
| oWG165 | TTAAGCTTTTATTTGTGTTCAAGGATATTTTT | plasmid construction |
| oWG166 | TTCCCGGGATGGAAAACGAAGTAAACGCAGGA | plasmid construction |
| oWG167 | TTCCCGGGAGAGTGCGGTGTTAGCATTCCCTA | plasmid construction |
| oWG168 | TTAAGCTTATGGTGAGCAAGGGCGAGGAGCTG | plasmid construction |
| oWG169 | TTCCCGGGAGCTCCAGCTCCCTTGTACAGCTCGT | plasmid construction |
| oWG186 | TTCCGCGGAAAGTACGGAAAGCGTGTTC | plasmid construction |
| oWG188 | TTCCGCGGCTAATCTGGTAGTGGAATGTA | plasmid construction |
| oWG190 | TTCCGCGGCTATTCAACAGTATCATCAAC | plasmid construction |
| oWG216 | TTGGATCCATGTCTACCCCAGCTGAAT | plasmid construction |
| oWG217 | TTGCGGCCGCTCATTTATCATCATCATCTTTGTAATCGATGTCGTGGTCCTTATAGTCACCATCATGATCTTTGTAATCAGCTCCAGCTCCATTGTAGAGATCATCATCGTCCCC | plasmid construction |
| oWG440 | CAATTACAGTCGAGCATGGTGAGCAAGGGC | plasmid construction |
| oWG441 | CTTCGTTTTCCATAGCTCCAGCTCCCTTGTACAGCTCGTC | plasmid construction |
| oWG442 | GACGAGCTGTACAAGGGAGCTGGAGCTATGGAAAACGAAG | plasmid construction |
| oWG443 | GCGGACTCTAGAGTTATTCAACCCATTACC | plasmid construction |
| oWG448 | CAATTACAGTCGAGCATGGTGAGCAAGGGC | plasmid construction |
| oWG449 | GCTGGGGTAGACATAGCTCCAGCTCCCTTGTACAGCTCGT | plasmid construction |
| oWG450 | ACGAGCTGTACAAGGGAGCTGGAGCTATGTCTACCCCAGC | plasmid construction |
| oWG451 | TTGCGGACTCTAGAGCTAATTGTAGAGATC | plasmid construction |
| oWG452 | GCTGGGGTAGACATAGCTCCAGCTCCCTTGTACAGCTCGTC | plasmid construction |
| oWG453 | GACGAGCTGTACAAGGGAGCTGGAGCTATGTCTACCCCAGC | plasmid construction |
| oWG454 | CAATTACAGTCGAGCATGAATCATTCAGCG | plasmid construction |
| oWG455 | TTGCGGACTCTAGAGTCAAGATCCACCAG | plasmid construction |
| F, Forward primer; R, Reverse primer | |  |

**Table S12. Genotyping primer combinations used in this study.**

| Mutants | Primers | Amplicon sizes (WT/mutant) in bp |
| --- | --- | --- |
| *wox1-2* | HBp130 +pZJ21+pZJ20 | 927/770 |
| *wox2-2* | HBp02+ERp16+TL13 | 693/500 |
| *wox2-4* | HBp01+pZJ31+TL43 | 851/500 |
| *wox3-2* | pZJ16+pZJ17+pZJ15 | 500/250 |
| *wox5-1* | TL63+TL64+TL43 | 210/460 |
| *cdc48a-1*/+ | oWG191+oWG192+oWG193 | 1067/667 |
| *cdc48a-2*/+ | oWG191+oWG195+oWG194 | 1112/784 |
| *cdc48a-3*/+ | oWG191+oWG196+oWG197 | 1124/721 |
| WT, wildtype. | | |

**Supplemental References**

Haecker A, Groß-Hardt R, Geiges B, Sarkar A, Breuninger H, Herrmann M, Laux T (2004) Expression dynamics of WOX genes mark cell fate decisions during early embryonic patterning in *Arabidopsis thaliana*. Development 131:657-668

Nelson BK, Cai X, Nebenfuhr A (2007) A multicolored set of in vivo organelle markers for co-localization studies in Arabidopsis and other plants. Plant J 51:1126-1136

Park S, Rancour DM, Bednarek SY (2008) In planta analysis of the cell cycle-dependent localization of AtCDC48A and its critical roles in cell division, expansion, and differentiation. Plant Physiol 148:246-258

Stolpe T, Susslin C, Marrocco K, Nick P, Kretsch T, Kircher S (2005) In planta analysis of protein-protein interactions related to light signaling by bimolecular fluorescence complementation. Protoplasma 226:137-146

Zhang Z, Tucker E, Hermann M, Laux T (2017) A Molecular Framework for the Embryonic Initiation of Shoot Meristem Stem Cells. Dev Cell 40:264-277 e264
